# The ghost of introduction past: Spatial and temporal variability in the genetic diversity of invasive smallmouth bass

**DOI:** 10.1111/eva.12652

**Published:** 2018-06-25

**Authors:** Genevieve Diedericks, Romina Henriques, Sophie von der Heyden, Olaf L. F. Weyl, Cang Hui

**Affiliations:** ^1^ Centre for Invasion Biology Department of Botany and Zoology Stellenbosch University Matieland Stellenbosch South Africa; ^2^ Evolutionary Genomics Group Department of Botany and Zoology Stellenbosch University Matieland Stellenbosch South Africa; ^3^ Section for Marine Living Resources National Institute of Aquatic Resources Technical University of Denmark Lyngby Denmark; ^4^ DST/NRF Research Chair in Inland Fisheries and Freshwater Ecology South African Institute for Aquatic Biodiversity (SAIAB) Grahamstown South Africa; ^5^ Centre for Invasion Biology South African Institute for Aquatic Biodiversity (SAIAB) Grahamstown South Africa; ^6^ Centre for Invasion Biology Department of Mathematical Sciences Stellenbosch University Matieland Stellenbosch South Africa; ^7^ Mathematical Biosciences Group African Institute for Mathematical Sciences Cape Town South Africa

**Keywords:** demographic history, genetic bottleneck, genetic diversity, historic DNA, invasive, multiple introductions, sampling design

## Abstract

Understanding the demographic history of introduced populations is essential for unravelling their invasive potential and adaptability to a novel environment. To this end, levels of genetic diversity within the native and invasive range of a species are often compared. Most studies, however, focus solely on contemporary samples, relying heavily on the premise that the historic population structure within the native range has been maintained over time. Here, we assess this assumption by conducting a three‐way comparison of the genetic diversity of native (historic and contemporary) and invasive (contemporary) smallmouth bass (*Micropterus dolomieu*) populations. Analyses of a total of 572 *M. dolomieu* samples, representing the contemporary invasive South African range, contemporary and historical native USA range (dating back to the 1930s when these fish were first introduced into South Africa), revealed that the historical native range had higher genetic diversity levels when compared to both contemporary native and invasive ranges. These results suggest that both contemporary populations experienced a recent genetic bottleneck. Furthermore, the invasive range displayed significant population structure, whereas both historical and contemporary native US populations revealed higher levels of admixture. Comparison of contemporary and historical samples showed both a historic introduction of *M. dolomieu* and a more recent introduction, thereby demonstrating that undocumented introductions of this species have occurred. Although multiple introductions might have contributed to the high levels of genetic diversity in the invaded range, we discuss alternative factors that may have been responsible for the elevated levels of genetic diversity and highlight the importance of incorporating historic specimens into demographic analyses.

## 1 INTRODUCTION

Understanding the demographic history of populations constitutes a fundamental aspect of evolutionary biology. Invasive species are particularly suitable for demographic analyses, as they frequently experience rapid alternations in levels of genetic diversity following introduction (Chown et al., 2015; Hui & Richardson, 2017; Lee, 2002; Rius & Darling, 2014; Roman & Darling, 2007). To this end, the assessment of genetic diversity has become essential for establishing the demographic and adaptive potential of populations in novel environments (Dlugosch, Anderson, Braasch, Cang, & Gillette, 2015; Prentis, Wilson, Dormontt, Richardson, & Lowe, 2008; Stapley, Santure, & Dennis, 2015; Zenni, Bailey, & Simberloff, 2014) and provides insight into the role that genetic variability plays in an organisms’ invasive success (Edelaar et al., 2015). Ultimately, this information allows predictions to be made on population viability, aiding in the development of an appropriate, species‐specific management strategy (Chown et al., 2015; Meyer et al., 2017; Prentis et al., 2008).

Numerous studies have attempted to assess the effects of invasion dynamics on genetic variation (e.g., founder effects, genetic bottlenecks, admixture, propagule pressure; Baker & Stebbins, 1965; Hui & Richardson, 2017; Mayr, 1963) by comparing populations in the native and invasive ranges (Kelly, Muirhead, Heath, & Macisaac, 2006; Kolbe et al., 2004; Naccarato, Dejarnette, & Allman, 2015; Rollins, Woolnough, Wilton, Sinclair, & Sherwin, 2009). These types of studies aid in unravelling the demographic history of the invasive species in question (Ficetola, Bonin, & Miaud, 2008; Gillis, Walters, Fernandes, & Hoffman, 2009; Gray et al., 2014; Neilson & Stepien, 2011). Yet, despite the wealth of specimens and information housed within Natural History collections, the majority of invasion studies to date have focussed exclusively on contemporary populations, thereby relying heavily on the premise that the historic population structure within the native range has been maintained over time.

Historic DNA serves as a valuable reference when examining contemporary genetic diversity (Bouzat, 2000; Dormontt et al., 2014; Guinand, Scribner, & Page, 2003; Lozier & Cameron, 2009), as it allows for the monitoring of temporal changes in genetic diversity across generations (Guinand et al., 2003; Sefc, Payne, & Sorenson, 2007). This temporal approach increases the chance of detecting subtle changes frequently overlooked by studies focussing only on contemporary data (Lozier & Cameron, 2009) and thus allows us to delineate the most likely invasion scenario (Gillis et al., 2009; Thompson et al., 2011; Van Kleunen, Weber, & Fischer, 2010) and reveal connectivity levels among invasive populations (Beneteau, Walter, Mandrak, & Heath, 2012; Funk, Garcia, Cortina, & Hill, 2011; Snyder & Stepien, 2017). This may be of particular importance in studies conducted on taxa for which there is a priori reason to suspect temporal fluctuations in genetic variation, such as highly exploited (and subsequently stocked) taxa or species often associated with human‐mediated dispersal. Hence, from an evolutionary perspective, the incorporation of historic DNA is therefore of fundamental importance.

Smallmouth bass, *Micropterus dolomieu* (Lacepèdé, 1802), presents a suitable model system to investigate variation in genetic diversity through space and time, as the species’ exploitation and subsequent stocking events within the native range are well documented (Long, Allen, Porak, & Suski, 2015), and its formal introduction history and subsequent spread into and throughout South Africa are well recorded (De Moor & Bruton, 1988). Twenty‐nine *M. dolomieu* specimens originating from broodstock collected in the Wheeling River, West Virginia, USA, were shipped from the Lewistown hatchery in Maryland, USA, to the Jonkershoek hatchery in South Africa in 1937 (De Moor & Bruton, 1988; Loppnow, Vascotto, & Venturelli, 2013; Powell, 1967). Here, they were reared and bred before being released into multiple water bodies across the country to provide opportunities for angling (De Moor & Bruton, 1988). Most of the documented stockings (De Moor & Bruton, 1988) occurred prior to the cessation of government support to stocking programs in the early 1990s (Ellender, Woodford, Weyl, & Cowx, 2014).

Considering that both the historical record and contemporary distributions of *M. dolomieu* in South Africa are well documented, this study aims to (a) assess the genetic differentiation and diversity within *M. dolomieu* populations in South Africa, (b) investigate how genetic diversity changed over time in both native and invasive ranges, and (c) assess the introduction history of *M. dolomieu* into South Africa. Given the small *M. dolomieu* founding population, we predict that the invasive South African range will have a lower genetic diversity when compared to the native (historic and contemporary) North American range due to a loss of alleles, as suggested by Dlugosch and Parker (2008). Furthermore, as heavily exploited species often experience genetic bottlenecks, leaving traces in the species’ genetic diversity (Pinsky & Palumbi, 2014), we predict that the genetic diversity will be lower in contemporary time when compared to historical samples in the native range.

## 2 MATERIALS AND METHODS

### 2.1 DNA collection and extraction from historical specimens

**Figure 1:**
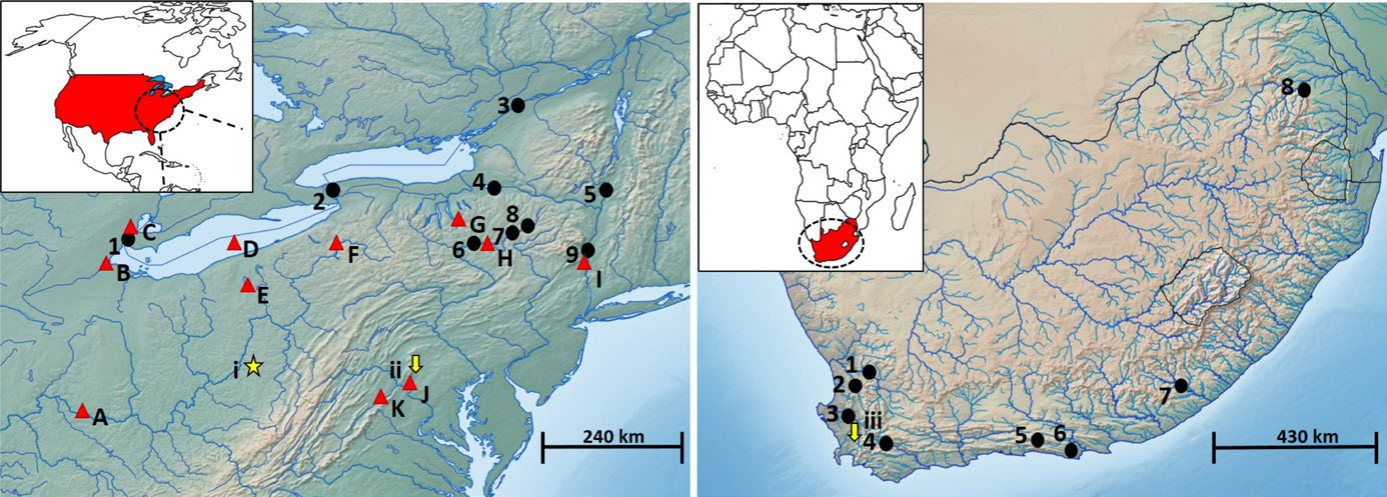
Map of native USA (left) and invasive SA (right) sampling localities. Letters A‐K denote historical sampling localities, while numbers denote contemporary sampling localities. All letters and numbers correspond to those used in Table 1. The location indicated by the star (i) represent the Wheeling River, while the downward‐facing arrows denote the (ii) Lewistown hatchery and (iii) Jonkershoek hatchery, respectively

Specimens representing the historical native range (Figure 1), corresponding to the approximate time of introduction into South Africa (1930–1941), were obtained from a host of collections housed at the Smithsonian National Museum of Natural History (NMNH), The Academy of Natural Sciences of Drexel University (ANSP), University of Michigan Museum of Zoology (UMMZ) and the Ohio State University Museum (OSUM) (Table 1; Appendix 1). In total, 53 formalin‐fixed specimens representing 11 drainage systems were obtained for genetic analyses (Table 1). These specimens represent a subset of the *M. dolomieu* genetic diversity that was present in the native range 20–25 generations ago (Barthel et al., 2008).

**Table 1:** An overview of the sampled populations from the contemporary invasive (CI), contemporary native (CN) and historical native (HN) ranges. Abbreviations correspond to those used in subsequent tables, text and Appendix1

	Native/invasive	State/province	Collection date	Sampled locality	Drainage system	Abbr. in Tables	*N*	Formaldehyde exposure	Material supplied By	Symbol on sampling map (Figure 1)
Historical specimens	Native	Ohio	1930	White Oak Creek	Ohio River	OH	3	Yes	OSUM	A
	Native	Ohio	1940; 1941	Auglaize River	Auglaize River	AU	5	Yes	OSUM	B
	Native	Michigan; Ontario	1934; 1935; 1940	Detroit River	Detroit River	DET	18	Yes	UMMZ	C
	Native	Ohio	1941	Lake Erie	Lake Erie	LE	3	Yes	OSUM	D
	Native	Ohio	1938	Mosquito Creek Lake	Mosquito Creek	MO	2	Yes	OSUM	E
	Native	New York	1937	Allegheny River	Allegheny River	AL	3	Yes	UMMZ	F
	Native	New York	1931	Fall Creek	Cayuga Lake, Etna	FC	2	Yes	UMMZ	G
	Native	New York	1935	Otselic River; Susquehanna River	Susquehanna River	SU	5	Yes	UMMZ	H
	Native	New York	1936	Rondout River	Hudson River	HUD	4	Yes	UMMZ	I
	Native	Maryland	1941	Monocacy River	Potomac River	PO	4	No	ANSP	J
	Native	Virginia; West Virginia	1933–1936	Shenandoah River	Shenandoah River	SH	4	Yes	NMNH	K
							**53**			
Contemporary Specimens	Native	Ontario	2013; 2014	Detroit River	Detroit River	DET	7	Yes	ROM	1
	Native	New York	2014	Niagra River	Niagra River	NIA	49	No	USA collectors	2
	Native	New York	2014	St Lawrence River	St Lawrence River	STL	55	No	USA collectors	3
	Native	New York	2015	Oneida Lake	Oneida River	ONEI	27	No	USA collectors	4
	Native	New York	2015	Saratoga Lake	Hudson River	SAR	10	No	USA collectors	5
	Native	New York	2015	Vestal; Susquehanna River	Susquehanna River	VES	14	No	USA collectors	6
	Native	New York	2015	Oneonta; Susquehanna River	Susquehanna River	ONEO	10	No	USA collectors	7
	Native	New York	2015	Lolliersville	Susquehanna River	LOL	20	No	USA collectors	8
	Native	New York	2014	Hudson River	Hudson River	HUD	21	No	USA collectors	9
							**213**			
	Invasive	Western Cape	2014	Doring River	Doring River	DO	38	No	Self‐collected	1
	Invasive	Western Cape	2014	Olifants River; Jan Dissels River	Olifants River	OL	44	No	Self‐collected	2
	Invasive	Western Cape	2014	Berg River	Berg River	BE	22	No	Self‐collected	3
	Invasive	Western Cape	2014	Breede River	Breede River	BR	43	No	Self‐collected	4
	Invasive	Eastern Cape	2014	Kouga River	Kouga River	KO	46	No	Self‐collected	5
	Invasive	Eastern Cape	2012	Krom River	Krom River	KR	15	No	SAIAB	6
	Invasive	Eastern Cape	2014	Rooikranz Dam	Buffalo River	BU	48	No	SAIAB	7
	Invasive	Mpumalanga	2014	Blyde Dam	Blyde River	MP	50	No	MPB	8
							**306**			

Genomic DNA was extracted from preserved muscle tissue (20–50 mg) in a room previously unexposed to fish DNA using sterilized equipment. Prior to each extraction, all equipment and surfaces were treated with 10% bleach to remove any potential contaminants. Pikor, Enfield, Cameron, and Lam (2011) showed that high‐quality DNA can be extracted from formalin‐fixed tissue if the samples are rehydrated with a series of ethanol washes prior to extraction. Thus, 500 μl of 100% ethanol was added to each tissue sample and vortexed vigorously for 30 s. The liquid was removed, and the process was repeated with 500 μl 70% ethanol, followed by 1,000 μl distilled water. Lastly, 1,000 μl distilled water was added to each sample and left to soak at 55°C for 5 days, vortexing the sample every 24 hr. Once rehydrated, the sample was moved to a dry Eppendorf tube before DNA extraction, using the QIAamp DNA FFPE tissue extraction kit (QIAGEN). In a recent review, Paireder et al. (2013) demonstrated that this kit consistently outcompeted other extraction methods when working with old (1820–1950), formalin‐fixed tissue. Apart from doubling the amount of proteinase K added to each sample (60 μl), extraction followed the manufacturers’ protocol. To break the formalin bonds, the samples were heated to 90°C for 1 hr before commencing with the wash steps. Lastly, to ensure the maximum elution of bound DNA, 10 μl elution buffer (warmed to 25.5°C) was added and left to “incubate” at room temperature for 5 min before centrifuging at 20,000 *g* for 1.5 min. This was repeated three times to yield a total DNA extraction volume of 30 μl. All DNA extractions were stored at −20°C.

### 2.2 DNA collection and extraction from contemporary specimens

Fresh tissue samples (muscle, liver, fin clippings) were derived from specimens collected by angling in both the native United States of America (USA) and Canada and the invasive South African (SA) ranges during the summer months of 2014 and 2015 (Figure 1). Collections in North America were led by a host of individuals and organizations based in the USA and Canada (see Acknowledgements). Nine localities rendering a total of 213 specimens were sampled from the same “broad” area represented by the historical samples to allow for direct genetic diversity comparisons (Table 1). Additional specimens collected in 2014 (*n* = 7; formalin fixed), representing the Detroit River, were obtained from the Royal Ontario Museum (ROM), Canada.

All SA specimens were euthanized with clove oil (CapeNature permit number 0056‐AAA043‐00004; Eastern Cape permit numbers CRO 165/14CR and CRO 166/14CR; Mpumalanga permit number MPB. 5498/2; Ethical clearance reference number SU‐ACUM14‐00011, University of Stellenbosch) before sampling a piece of tissue. Tissue samples were stored in 70% ethanol for subsequent DNA extraction. Additional specimens (*n* = 63) were obtained from the South African Institute for Aquatic Biodiversity (SAIAB), Grahamstown, South Africa, rendering a total sample size of 306 specimens representing eight river systems (Table 1; Appendix 1). DNA was extracted from each contemporary specimen (USA & SA) using the NucleoSpin Tissue extraction (gDNA) kit (MACHEREY‐NAGEL, Separations, Cape Town, South Africa) following the manufacturers’ protocol. All DNA extractions were stored at −20°C.

### 2.3 Historical and contemporary DNA amplification

To corroborate the morphological identification of the contemporary collected specimens and assess genetic diversity and demographic history of both native and invasive populations, two partial mitochondrial DNA (mtDNA) gene regions, namely cytochrome b (cytb) and control region (CR), were amplified for all the contemporary samples (*n* = 519). This was not possible for the historical samples due to the limited availability of tissue and the degraded nature of the DNA. A standard 25 μl mastermix was prepared for both mtDNA polymerase chain reactions (PCRs). The internal cytb primers, basscytbf1 (5′‐CAC CCC TAC TTC TCC TAC AAA GA‐3′) and basscytbr1 (5′‐AAG GCR AAG CGG GTG AGG G‐3′; Near, Kassler, Koppelman, Dillman, & Philipp, 2003), were used to amplify the cytb fragment. The primer set CB3R‐L (5′‐CATATTAAACCCGAATGATATTT‐3′; Palumbi, 1996) and HN20‐R (5′‐GTGCTTATGCTTTAGTTAAGC‐3′; Bernatchez & Danzmann, 1993) was used to amplify the CR. Both PCR reactions followed the authors’ protocols. All PCR products were visualized through gel electrophoresis before being sequenced (ABI 3730 XL DNA Analyzer, Applied Biosystems, CAF, Stellenbosch, South Africa). Chromatographs were visually inspected and aligned in Geneious^®^ 10.0.2 (Biomatters, Auckland, New Zealand).

Fifteen microsatellite loci, designed for both species‐ and genus‐level amplification, were selected from published literature (Supporting Information Table S1). Of these, only 11 loci (eight species‐specific: Mdo3, Mdo4, Mdo5, Mdo7, Mdo8, Mdo9, Mdo10, Mdo11—Malloy, Den Bussche, Jr, Coughlin, & Echelle, 2000; and three genus‐specific: Lma21—Colbourne, Neff, Wright, & Gross, 1996; Lma102, Lma117—Neff, Fu, & Gross, 1999) were successfully amplified. As Lma102 and Lma117 were not polymorphic for a subset of specimens, they were excluded; therefore, nine polymorphic loci were used in the present study (Supporting Information Table S1). Three multiplex reactions were performed using the KAPA2G™ Fast Multiplex PCR Kit (KapaBiosystems, Cape Town, South Africa).

The same nine microsatellite loci were amplified for the historic samples, following the amplification procedure used for the contemporary DNA, but due to the degraded nature of the DNA, this did not yield results. Thus, the resulting PCR products for each multiplex were diluted with distilled water to obtain a 1/10 PCR product which, in turn, served as template in the subsequent PCR. To ensure amplification and to avoid the overestimation of genetic diversity often associated with the amplification of ancient‐ and formalin‐fixed DNA (Buchan, Archie, Van Horn, Moss, & Alberts, 2005; Sefc et al., 2007), historical samples were amplified twice for each microsatellite locus. All microsatellite genotyping was performed on an ABI 3730 XL DNA Analyzer (Applied Biosystems, CAF, Stellenbosch, South Africa), using LIZ as an internal size marker, and scoring was conducted in Geneious^®^ 10.0.2 (Biomatters, Auckland, New Zealand). To ensure accurate scoring, reference individuals previously scored were used as positive controls. Historical specimens were scored blindly (i.e., specimen name removed) and repeated three times to ensure accuracy and consistency. Where scoring inconsistencies were observed (historical specimens) and more than three loci could not be scored (for both historical and contemporary specimens), the entire specimen was removed from the data set and excluded from the study. Similarly, as one microsatellite locus, Mdo8, did not amplify for the majority of historical samples, it was removed from the historical data set entirely. Thus, nine microsatellite loci were analysed for the contemporary data set, but only eight microsatellite loci were analysed for the historical data set.

### 2.4 Contemporary mtDNA analyses

To assess genetic diversity levels in both the contemporary native (USA—CN) and invasive (SA—CI) ranges, the number of haplotypes (*H*), haplotype diversity (*h*) and nucleotide diversity (π) were calculated for each sample site. The population history for *M. dolomieu* in both ranges was examined using Fu's *Fs* (Fu, 1997) and Tajima's *D* (Tajima, 1989). Assessment of genetic population structure was conducted combining both native and invasive contemporary data sets for each gene fragment. Pairwise *F*_ST_ values were calculated and a hierarchical analysis of molecular variance (AMOVA) conducted to determine the amount of population subdivision among sampled localities. All analyses were conducted in ARLEQUIN 3.5.2.2 (Excoffier & Lischer, 2010), with statistical significance assessed with 10,000 permutations.

### 2.5 Contemporary and historical microsatellite analyses

All microsatellite loci were assessed for linkage disequilibrium and deviations from Hardy–Weinberg equilibrium (HWE) in Genepop 4.2.1 (Rousset, 2008), with statistical significance assessed after 10,000 iterations. The Bonferroni method was used to correct for multiple comparisons (Rice, 1989). Amplification errors associated with large allele dropout and stuttering were assessed with MICROCHECKER 2.2.3 (Van Oosterhout, Weetman, & Hutchinson, 2006). As most of the populations were found to not comply with HWE assumptions, FreeNA 1.2 (Chapuis & Estoup, 2007) was used to check for the presence of null alleles using the EM algorithm (Dempster, Laird, & Rubin, 1977). Intraspecific and within‐population genetic diversity levels were assessed as number of alleles (Na), allelic richness (AR), observed (*H*_O_) and expected heterozygosity (*H*_E_), and Wright's inbreeding coefficient (*F*_IS_), as implemented in FSTAT 2.9.3.2 (Goudet, 1995), Genepop 4.2 (Rousset, 2008), HP‐Rare 1.1 (Kalinowski, 2005) and ARLEQUIN 3.5.2.2 (Excoffier & Lischer, 2010). Statistical significance of *F*_IS_ was assessed after 1,000 permutations in FSTAT 2.9.3.2 (Goudet, 1995). Allelic richness (AR) was calculated using HP‐Rare 1.1 (Kalinowski, 2005), correcting for sample size disparity through rarefaction analysis. Analyses were conducted per population for the two contemporary data sets, but due to the small sample size for most of the historical localities (Table 1), these were grouped (= MUS) to obtain the genetic diversity indices.

Multiple approaches were employed to investigate the population structuring and genetic connectivity among (contemporary and historical) populations. As only eight loci were successfully amplified for the historical native (HN) specimens, all comparative analyses incorporating the historical samples only compared the eight loci, while contemporary SA–USA comparisons encompassed nine loci. First, to determine whether there was a difference in observed heterozygosity (*H*_O_) between the three groups (CI, CN, HN), an analysis of variance (ANOVA) was conducted in spss statistics 20.0.0 (SPSS Inc., Chicago, IL, USA), with loci selected as random factors. Subsequently, a Bonferroni post hoc test was used to further assess the differences between groups. In addition, a stacked bar graph was constructed to visualize the variation among localities and loci. Second, Weir's (1996) *F*_ST_ was employed to assess the genetic differentiation among sampled localities using FreeNA 1.2 (Chapuis & Estoup, 2007). FreeNA, employing the *ENA* correction method (Chapuis & Estoup, 2007), was chosen as it has been shown to correctly estimate *F*_ST_ values in the presence of null alleles (detected in the previous analysis; Chapuis & Estoup, 2007). A jackknife approach with 1,000 bootstrap replicates was employed to assess statistical significance (Chapuis & Estoup, 2007). Next, BOTTLENECK 1.2.02 (Piry, Luikart, & Cornuet, 1999) was used to test the prediction that both contemporary populations (CI and CN) experienced a recent genetic bottleneck. Populations that have undergone a genetic bottleneck are often associated with a loss of (rare) alleles and display elevated levels of heterozygosity when compared to stable populations (Piry et al., 1999). Thus, significant heterozygote excess was evaluated for each of the three groups using a Wilcoxon rank test (10,000 iterations) for two mutational models often associated with microsatellite evolution: the two‐phase mutation model (TPM) and the infinite alleles model (IAM).

To investigate the genetic associations within each of the three groups as well as among them, without being influenced by the lack of HWE or the presence of null alleles, a principal component analysis (PCA) using microsatellite allelic frequencies was conducted in the R package Adegenet 1.3.1 (Jombart & Ahmed, 2011). Next, we used STRUCTURE 2.3.4 (Pritchard, Stephens, & Donnelly, 2000) to (a) identify and visualize the population structure within each of the three groups (CI, CN and HN), (b) compare overlapping populations from the historical and contemporary native range and (c) search for a potential source population from where the invasive South African stocks originated. Four STRUCTURE analyses (each group independently followed by an analysis combining CI, CN and HN) were conducted using the admixture model with correlated allele frequencies, allowing each individual to be allocated to multiple clusters as determined by its genotype frequency. Five replicate runs were conducted for each *K* (1 < *K *< 15). Runs were conducted using an initial burn‐in of 75,000 Markov chain Monte Carlo (MCMC) generations, followed by 350,000 MCMC steps. STRUCTURE HARVESTER 0.6.94 (Earl & vonHoldt, 2012) was used to determine the most probable *K* following the Evanno method (Evanno, Regnaut, & Goudet, 2005), before using CLUMPP 1.1.2 (Jakobsson & Rosenberg, 2007) to compile the five replicate runs for the most likely *K*. DISTRUCT 1.1 (Rosenberg, 2004) was used to visualize the composite assignments.

At last, we performed an approximate Bayesian computation (ABC) on the microsatellite data set to determine whether the invasive South African *M. dolomieu* populations originated from a single introduction event from the USA as stated by the historical records, using DIYABC 2.1.0 (Cornuet et al., 2014). As null alleles were only observed in one locus (Mdo9) of the HN data set, all loci and populations were included. Sampled localities were pooled into three groups (CI, CN and HN), and six simple, yet competing, introduction scenarios were generated under a coalescent framework (Figure 5: 1–6), to focus the computational efforts on probable introduction scenarios rather than an exhaustive list of possibilities (see Appendix 2 for detailed introduction scenarios). As the STRUCTURE results revealed that a subsample of the invasive South African *M. dolomieu* individuals (CI_S_) were more closely related to the historic native samples than to the remaining SA individuals (CI) (predominantly individuals from populations BE and OL; Figure 4: b), we simulated nine additional scenarios to test the theory of multiple introductions (Figure 5: A–I; Appendix 2). At last, as suggested by Guillemaud, Beaumont, Ciosi, Cornuet, and Estoup (2010), three supplementary scenarios were simulated to determine whether the two SA groupings (CI and CI_S_) originated from (a) a single serial introduction from the source population (CN + HN), (b) two independent introduction events from the same source or (c) an unsampled source population (Figure 5: i–iii; Appendix 2). To prevent overparameterization, parameters were specified according to the program guidelines (Cornuet et al., 2014). First, we performed a pre‐evaluation of the data set to ensure that at least one scenario and its associated priors could generate simulated data sets similar to that of the observed. This was accomplished by simulating 100,000 data sets and comparing summary statistics for both simulated single‐sample (i.e., mean number of alleles, genetic diversity and allele size variance across loci) and two‐sample statistics (i.e., mean genetic diversity, number of alleles, allele size variance, mean index of classification, shared allele distance, distance between samples and *F*_ST_) to the observed data (Cornuet et al., 2014). As the mean *M* index across loci (Garza & Williamson, 2001) was initially developed with conservation planning in mind, this statistic does not perform well with small, unequal sampling sizes and small starting population sizes (Garza & Williamson, 2001). Hence, it was excluded from the summary statistics used in the current analyses. Next, we simulated 10^6^ data sets per scenario before calculating the posterior probability (PP) for each. Scenarios were subsequently compared through a logistic regression, which was conducted on the linear discriminant analysis components (Cornuet et al., 2014). Each scenarios error rate was evaluated by generating 100 pseudo‐observed data sets, using parameter values obtained from one of the scenarios (e.g., scenario 1). The type I error rate was determined by counting the number of times the PPs were higher for any scenario other than the chosen scenario, divided by the number of pseudo‐observed data sets (i.e., 100), while the type II error rate was calculated by counting the number of pseudo‐observed data sets that unrightfully received the highest PP support (Cornuet, Ravigne, & Estoup, 2010).

## 3 RESULTS

### 3.1 Contemporary mtDNA analyses

A total of 292 *M. dolomieu* specimens collected from eight river systems in the invasive SA range (CI) were successfully sequenced for 306 bp of cytb and 979 bp of CR, while the nine native USA (CN) localities yielded a total of 209 and 174 successfully sequenced *M. dolomieu* specimens for cytb and CR, respectively. Both cytb and CR rendered fewer haplotypes for the CN range when compared to the CI range, but similar haplotype and nucleotide diversity levels were observed (Table 2). Overall, high haplotype and low nucleotide diversity levels were observed for both native (cytb: *h *=* *0.976 ± 0.005, π = 0.051 ± 0.025; CR: *h *=* *0.977 ± 0.007, π = 0.044 ± 0.021) and invasive (cytb: *h *=* *0.967 ± 0.007, π = 0.087 ± 0.043; CR: *h *=* *0.985 ± 0.003, π = 0.039 ± 0.019) populations, but differed between sampling localities and gene fragment (Table 2). In particular, overall nucleotide diversity was higher for cytb in the CI populations (Table 2). Significant deviations from neutrality were observed for Tajima's *D* and Fu's *Fs* in both native and invasive range and both gene fragments (Table 2). Pairwise *F*_ST_ measures revealed two significantly differentiated groupings: CI and CN (Supporting Information Table S2), with comparisons between localities from the two groups ranging from *F*_ST_ = 0.013 to *F*_ST_ = 0.172 (both *p *<* *0.05) for cytb (DO—SAR and KO—VES) and *F*_ST_ = 0.013 to *F*_ST_ = 0.125 (both *p *<* *0.05) for CR (KR—NIA and BE—LOL; Supporting Information Table S2). With regard to the cytb gene fragment, the CN DET population was not significantly different from any of the CI populations, except KO. Similarly, for the CR, the CN populations ONEO and SAR were not significantly different from the majority of CI populations (Supporting Information Table S2). Significant within grouping, differentiation (though markedly less so for the USA cytb) was also observed in both cytb and CR (Supporting Information Table S2). The AMOVA results revealed that the largest proportion of genetic variation (cytb: 94.79%; CR: 95.79%) was distributed within each population, with very little variation observed between the groups (cytb: 2.15%; CR: 1.58%), as well as among populations within groups (cytb: 3.06%; CR: 2.26%). All variance components were significantly different from 0 (*p *<* *0.001).

**Table 2:** Genetic diversity indices (haplotype (***h***) and nucleotide (**π**)) and neutrality tests (Tajima's ***D*** and Fu's ***Fs***) for the contemporary invasive (CI) and contemporary native (CN) ranges, based on mtDNA cytb and CR. Sample size is denoted by **n**, while **H** refers to the number of haplotypes. Statistically significant results (*p *<* *0.05) are indicted in bold

	Cytochrome b (cytb)						Control region (CR)					
	*n*	*H*	*h*	π	*D*	*Fs*	*n*	*H*	*h*	π	*D*	*Fs*
Contemporary invasive SA localities												
BE	20	16	0.963 ± 0.033	0.066 ± 0.034	**−1.682**	−1.758	21	14	0.867 ± 0.074	0.088 ± 0.044	**−2.277**	6.160
BR	42	33	0.976 ± 0.014	0.061 ± 0.031	−1.295	**−9.88**	43	33	0.981 ± 0.011	0.036 ± 0.018	**−2.011**	−4.340
BU	47	30	0.965 ± 0.013	0.061 ± 0.031	**−2.004**	−4.574	47	35	0.984 ± 0.008	0.020 ± 0.010	**−2.594**	**−10.918**
DO	35	30	0.987 ± 0.012	0.263 ± 0.129	0.314	−1.295	36	30	0.979 ± 0.016	0.084 ± 0.041	**−2.537**	0.321
KO	46	24	0.756 ± 0.071	0.044 ± 0.022	**−2.310**	−2.777	45	36	0.984 ± 0.010	0.013 ± 0.007	**−1.71**	**−21.924**
KR	14	9	0.835 ± 0.101	0.050 ± 0.027	**−1.768**	0.833	15	15	1.000 ± 0.024	0.046 ± 0.024	**−2.047**	−2.642
MP	45	37	0.987 ± 0.009	0.071 ± 0.036	−0.257	**−11.881**	45	31	0.942 ± 0.024	0.063 ± 0.031	**−2.646**	0.974
OL	43	24	0.947 ± 0.020	0.033 ± 0.017	**−2.071**	−5.458	40	17	0.906 ± 0.029	0.045 ± 0.022	**−1.603**	8.417
Overall	292	176	0.967 ± 0.007	0.087 ± 0.043	**−1.899**	**−23.547**	292	179	0.985 ± 0.003	0.039 ± 0.019	**−2.717**	**−23.604**
Contemporary native USA localities												
DET	7	7	1.000 ± 0.076	0.144 ± 0.083	0.767	−0.226	—	—	—	—	—	—
HUD	20	15	0.968 ± 0.025	0.050 ± 0.026	**−2.140**	−1.675	17	17	1.000 ± 0.020	0.134 ± 0.068	0.692	−1.145
LOL	20	16	0.974 ± 0.025	0.040 ± 0.021	**−1.940**	−3.662	20	13	0.884 ± 0.067	0.001 ± 0.001	−1.174	**−15.968**
NIA	48	31	0.957 ± 0.018	0.032 ± 0.017	**−2.445**	**−12.403**	38	28	0.976 ± 0.014	0.011 ± 0.006	**−2.157**	**−13.583**
ONEI	30	26	0.989 ± 0.013	0.022 ± 0.012	**−1.545**	**−20.166**	18	17	0.994 ± 0.021	0.082 ± 0.042	**−2.389**	−0.867
ONEO	10	8	0.956 ± 0.059	0.156 ± 0.084	−0.689	2.782	10	10	1.000 ± 0.045	0.012 ± 0.007	**−1.575**	**−4.188**
SAR	13	12	0.987 ± 0.035	0.030 ± 0.017	−0.615	**−4.471**	7	7	1.000 ± 0.076	0.301 ± 0.169	**−1.806**	2.179
STL	47	34	0.966 ± 0.017	0.032 ± 0.017	−0.829	**−18.178**	51	32	0.942 ± 0.023	0.002 ± 0.001	**−1.960**	**−28.464**
VES	14	10	0.923 ± 0.060	0.022 ± 0.012	**−1.950**	−2.114	13	10	0.962 ± 0.041	0.059 ± 0.031	−1.418	2.703
Overall	209	116	0.976 ± 0.005	0.051 ± 0.025	**−2.191**	**−23.870**	174	117	0.977 ± 0.007	0.044 ± 0.021	**−1.829**	**−23.756**

### 3.2 Contemporary and historical microsatellite analyses

A total of 519 contemporary sampled specimens, representing both invasive (*n* = 306; eight localities) and native (*n* = 213; nine localities) ranges, were successfully genotyped for nine microsatellite loci, while 53 museum samples, representing 11 localities within the historical native range, were successfully genotyped for eight microsatellite loci. Neither of the three groups (CI, CN and HN) displayed amplification errors (i.e., large allele dropout, stuttering), nor did any loci exhibit linkage disequilibrium. FreeNA (Chapuis & Estoup, 2007) revealed the presence of null alleles in microsatellite Mdo9 within the HN samples, but this was not the case for either of the contemporary groups. Deviations from HWE were observed in two CI populations (BE and OL) as well as the HN population (*F*_IS_: BE = 0.26, OL = 0.17, MUS = 0.43; Supporting Information Table S3). Further inspection revealed that this deviation was due to a heterozygote deficit within each of the three populations, suggesting the presence of a Wahlund effect (Wahlund, 1928; Waples, 2014), albeit negligible (Guillemaud et al., 2015; Lye, Lepais, & Goulson, 2011). Hence, all further analyses were conducted on the complete data set. The number of alleles (Na) and allelic richness (AR) were consistently higher in the HN data set, and similar between the two contemporary data sets: HN AR = 4.25, CI AR = 1.79–3.15, CN AR = 2.17–2.69 (Supporting Information Table S3). Multilocus genetic diversity (observed heterozygosity, *H*_O_) ranged from 0.39 (ONEI) to 0.59 (DET), while levels of expected heterozygosity (*H*_E_) ranged from 0.35 (MP) to 0.73 (MUS) across all loci.

**Figure 2:**
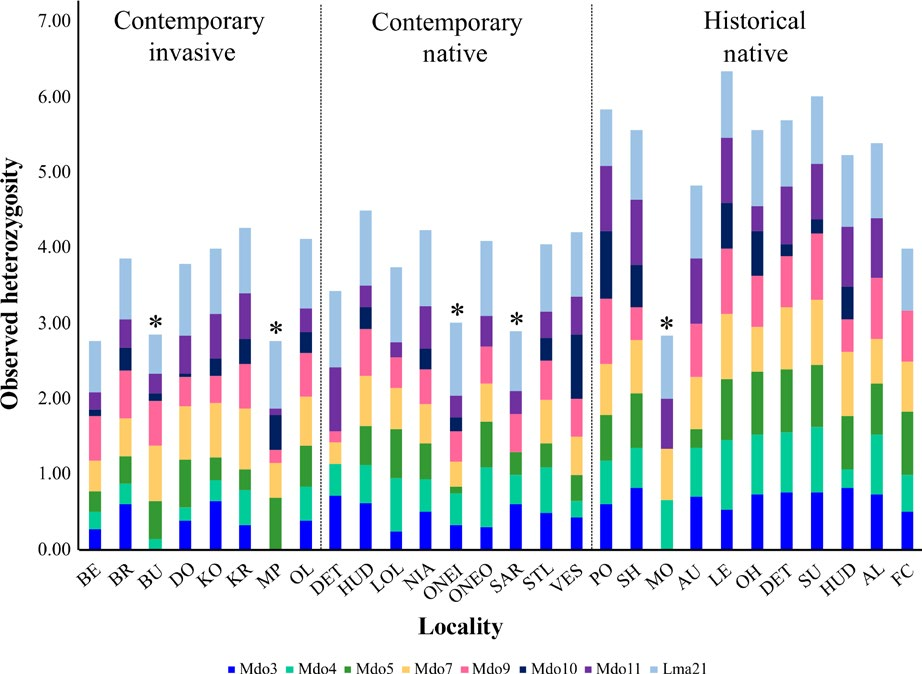
A stacked bar graph representing the variation in observed heterozygosity (*H*_O_) among populations and loci between the three groups (CI—contemporary invasive SA, CN—contemporary native USA, HN—historical native USA). Reservoirs (excluding Lake Erie (LE)) are indicated with an asterisk (*)

There was substantial variation in observed heterozygosity (*H*_O_) among populations and loci, with reservoirs (catchment size <5,000 km^2^) consistently displaying lower levels of *H*_O_ (Figure 2, Supporting Information Figure S2). Moreover, the ANOVA revealed significant differences in *H*_O_ between the three groups (*F*_2,214_ = 22.90, *p *= <0.001), with *H*_O_ being higher for HN compared to both contemporary groups (Bonferroni *post hoc* test *p *<* *0.001). A significant marker effect (*F*_7,214_ = 19.82, *p *<* *0.001) was, however, observed. Overall, *F*_ST_ among HN samples was significantly low (*F*_ST_ = 0.013; *p *<* *0.05), but this was not so for the CI (*F*_ST_ = 0.211; *p *<* *0.05) and CN (*F*_ST_ = 0.091; *p *<* *0.05) populations. Likewise, pairwise *F*_ST_ values revealed significant population differentiation among CI populations, ranging from *F*_ST_ = 0.066–0.469 (DO—KO and BE—MP), with similar results being observed when comparing populations across all three groups, that is, CI, CN and HN (*F*_ST_ = 0.123–0.537; MP—SAR and OL—MUS; Supporting Information Table S4). In contrast, CN populations displayed significantly less population differentiation among sampled localities within this group (*F*_ST_ = 0.072–0.129; LOL—NIA and SAR—STL; Supporting Information Table S4). As predicted, the Wilcoxon rank test revealed a significant excess of heterozygotes for both CI and CN under the IAM model (*p* = 0.002 and *p *=* *0.010, respectively), but this was not the case under the TPM model (CI: *p *=* *0.230; CN: *p *=* *0.473). Similarly, no significant excess of heterozygotes was detected for the HN population (IAM: *p *=* *0.473; TPM: *p *=* *0.998).

**Figure 3:**
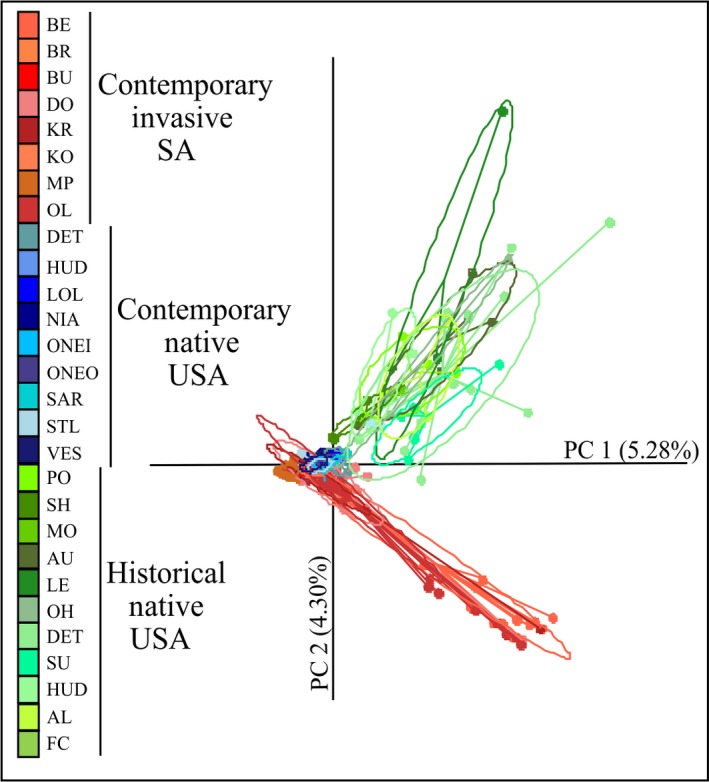
Principal component analysis (PCA) conducted on the combined microsatellite genotypes for the three groups (i.e., CI—contemporary invasive SA, CN—contemporary native USA, HN—historical native USA). Each dot represents a genotyped individual, and colours correspond to sampled localities. Variance explained in parentheses

**Figure 4:**
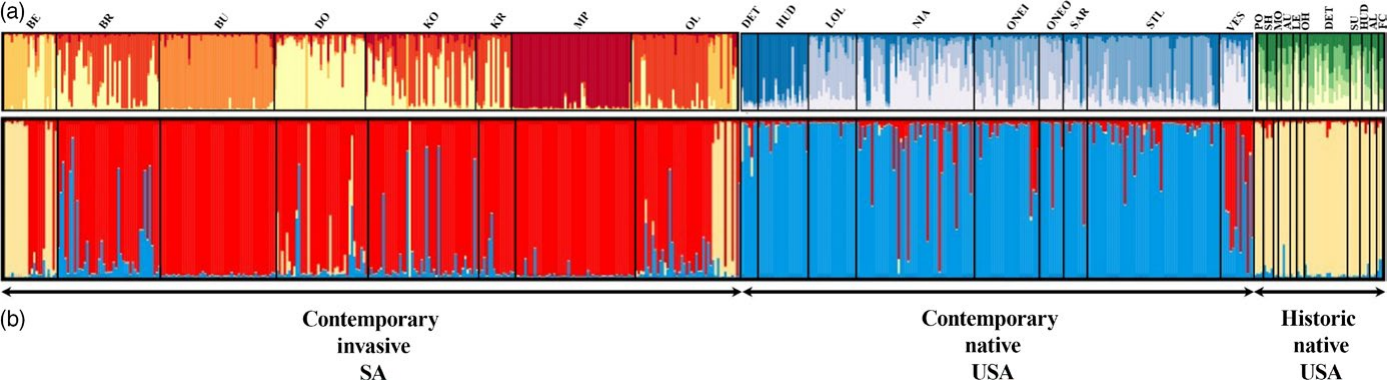
STRUCTURE plots representing the population structure within (a) each of the three groups (CI—contemporary invasive SA, CN—contemporary native USA, HN—historical native USA) when ran independently, and (b) population structure for all localities combined into a single run. Each genotyped individual is represented by a vertical line, with each lines’ colour proportional to the cluster membership of the individual

The principal component analysis (PCA), based on allelic frequencies, revealed two distinct groups along the first two axes: the first comprising both CN and CI populations and the second comprising the HN populations (Figure 3). Limited genetic associations between the two groups were observed. The Bayesian clustering analyses conducted in STRUCTURE revealed population substructuring within the CI localities, with Delta K (Evanno et al., 2005) retrieving *K *=* *5 as the most probable number of clusters (Figure 4a). Both CI reservoirs (BU and MP) were represented by their own cluster and showed very little population variation, corroborating the genetic diversity results (Figure 2; Supporting Information Table S3). The remaining six CI populations, however, displayed substantial levels of admixture, in particular localities BE and OL (Figure 4a). The CN populations exhibited high levels of population admixture indicative of shallow population differentiation, with Delta K revealing the most probable *K *=* *4 (Figure 4a). Similar levels of admixture and Delta K (*K *=* *4) were obtained for the HN populations (Figure 4a). To determine the most probable source population of the CI populations, all 28 localities were combined (Figure 4b). Delta K revealed the most probable number of clusters to be *K *=* *3, with each cluster representing a group, although admixture between the two contemporary groups was observed. Interestingly, a subset of individuals within the CI localities BE and OL (and to a lesser extent DO and KO) shared a cluster with HN, but this was not the case for any of the CN populations, despite overlapping sampling localities (DET, HUD, Susquehanna River: LOL, ONEO, VES, SU; Table 1; Figure 4b).

**Figure 5:**
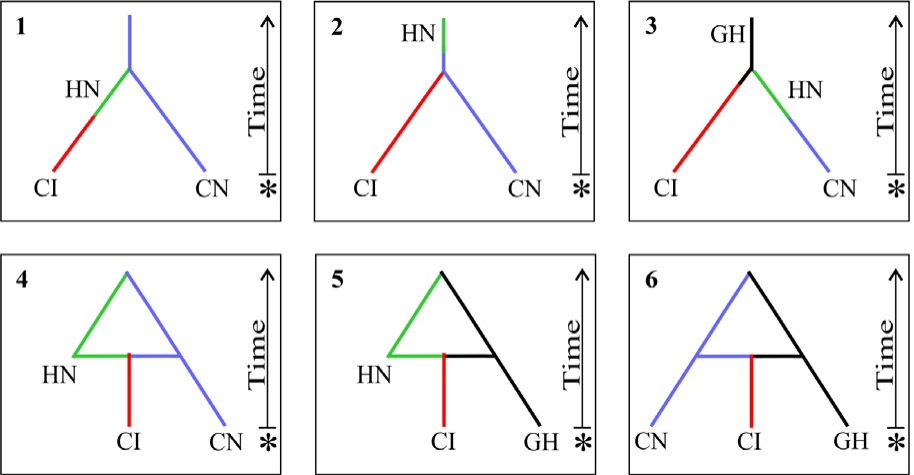
Probable introduction scenarios tested with approximate Bayesian computation as implemented in DIYABC. CI—contemporary invasive SA, CN—contemporary native USA, HN—historical native USA, GH—unsampled ghost population. The arrow indicates time expressed in generations (not to scale), with the present indicated with an asterisk

The ABC analysis consistently supported the notion of a more recent introduction. The first set of scenarios tested (Scenarios 1–6; Figure 5) revealed that Scenario 2 had the highest posterior probability (Supporting Information Table S5). The second set of analyses (Scenario A–I; Supporting Information Figure S1) supported both Scenarios C and F (Supporting Information Table S5). The third set of simulations (Scenarios i–iii; Supporting Information Figure S1), where we tested for a single versus multiple introductions from a single source or an unsampled source population, was inconclusive. Scenario iii (unsampled source population) did, however, marginally receive the most support (Supporting Information Table S5). Type I and Type II error rates were marginally low for the first two sets of simulations conducted (Supporting Information Table S5), but not for the third simulation (Supporting Information Table S5).

## 4 DISCUSSION

Numerous studies have compared genetic diversity levels across native and invasive ranges in an attempt to reconstruct the invasion history of invasive species (reviewed in Dlugosch & Parker, 2008; Lee, Patel, Conlan, Wainwright, & Hipkin, 2004; Novak & Mack, 2005; Rius & Darling, 2014; Roman & Darling, 2007), yet most of these studies only utilize contemporary genetic specimens. This, however, does not account for allele frequency shifts and assumes that the contemporary population structure within the native range would correspond to that of the historically native population. Using *M. dolomieu* as a study organism and incorporating both historical and contemporary native and invaded range samples, our results reveal that genetic diversity and population dynamics can indeed differ across both spatial and temporal scales.

### 4.1 Genetic diversity through space and time

Elevated levels of genetic diversity were observed in the contemporary invasive (CI) range when compared to the contemporary native (CN) range, contradicting the general assumption that genetic diversity is lower in recently invaded ranges than in long‐established native populations. However, when comparing all three groups, the historical native (HN) range displayed the highest levels of heterozygosity, number of alleles (Na) and allelic richness (AR). Although this might have resulted from a statistical artefact due to the smaller sample size for the HN range, similar findings were previously reported for Atlantic salmon (*Salmo salar*; Nielsen, Hansen, & Loeschcke, 1997). The authors observed a significant decrease in Na for the contemporary population when compared to samples collected 60 years before, likely due to a recent genetic bottleneck. Our results support this proposition, as the CN population exhibited high haplotype, but low nucleotide genetic diversity, as well as significantly negative Tajima's *D* and Fu's *Fs* levels, all of which are commonly observed in a population that had undergone a genetic bottleneck before experiencing expansion (Grant & Bowen, 1998). Moreover, the lack of population structure in the CN range, as well as low AR and Na, further supports this notion. Strong and sustained declines in population size, such as the ones experienced by commercially exploited species, are known to leave signatures in the genetic diversity of species, in particular by reducing Na and AR (Pinsky & Palumbi, 2014). Thus, the observed contemporary population dynamics of *M. dolomieu* in its native range might have resulted from the interaction between overfishing and restocking events during the last two centuries (Long et al., 2015). *Micropterus dolomieu* has been harvested both commercially and recreationally since the 1800s and has experienced several population declines and even extirpations in some localities (Marsh, 1867). This led the US government to start breeding programmes and enforce stricter regulations on fishing in the 1870s (Long et al., 2015). In 1903 alone, ~500,000 juvenile black bass were released into waterbodies across the USA (Bowers, 1905; Long et al., 2015; Loppnow et al., 2013). Concomitant fluctuations in population sizes are likely to have left genomic signatures and contributed to the observed elevated admixture in CN populations, as reintroductions were conducted without consideration for genetic population structure in *M. dolomieu*. Similar findings have been reported for another exploited freshwater species, the brook charr (*Silvanus fontinalis*), with individual admixture levels increasing with stocking intensity (Lamaze, Sauvage, Marie, Garant, & Bernatchez, 2012; Marie, Bernatchez, & Garant, 2010).

### 4.2 Population substructuring in an invaded range

Elevated levels of genetic diversity are, however, not uncommon in invasive species in a novel invaded range and are often attributed to multiple introductions and/or population mixture (see Rius & Darling, 2014 for a comprehensive review). The results from the STRUCTURE analyses appear to contradict the historical records stating that invasive South African *M. dolomieu* populations originate from a single introductory event from the USA in 1937. A genetic cluster encompassing samples from the Berg (BE: *n* = 14), Olifants (OL: *n* = 7), Doring (DO: *n* = 2), and Kouga (KO: *n* = 1) Rivers suggests shared ancestry with the HN samples, but the remainder of the invasive South African populations belong to four additional clusters, hinting at the idea of multiple introductions. The ABC results support this notion, as the best‐fit scenario suggested a second, more recent, introduction from North America (Scenario 2). Furthermore, when considering the invasive South African individuals associated with the HN STRUCTURE cluster as a separate South African population (CI_S_), the ABC analyses supported the STRUCTURE results and suggested at least two introductions: one coinciding with the recorded historic introduction and at least one more recent introduction. Indeed, the observed admixture between CI and CN suggests that the more recent introduction also originated from the USA. Unexpectedly, no support was obtained for either scenario examining single versus multiple introductions from a single source (Scenarios i and ii), nor any scenario postulating admixture (Scenarios 4, 5, 6). This may be due to several factors, such as the unequal sample sizes between HN and CI/CN range, the simplicity of the ABC models, or perhaps it could be ascribed to the fact that the HN population was not in HWE. Furthermore, the presence of a temporal Wahlund effect within the HN range, likely due to the pooling of multiple populations, may too have decreased the accuracy of the ABC results. Although our results support the notion of multiple introductions, this should be interpreted with caution as several factors may be responsible for this pattern, including an unsampled source population, postinvasion genetic drift, insufficient marker resolution and admixture in the source population (Chown et al., 2015; Gray et al., 2014). Given that hatcheries make use of artificial selection techniques to enhance species production and abundance (e.g., Aprahamian, Smith, McGinnity, McKelvey, & Taylor, 2003; Lamaze et al., 2012), it is possible that the introduced *M. dolomieu* were of admixed or hybrid origin, as has been reported for stockings of *S. fontinalis* (Cooper, Miller, & Kapuscinski, 2010; Lamaze et al., 2012; Sloss, Jennings, Franckowiak, & Pratt, 2008).

Invasive species capable of harbouring large, genetically diverse source populations are thought to make better invaders (Gaither, Bowen, & Toonen, 2013), as they are equipped with higher adaptive potential (Dlugosch, 2006; Lavergne & Molofsky, 2007; Wellband & Heath, 2017). Within the invasive South African range, *M. dolomieu* experiences an array of climatic conditions with fluctuating rainfall and temperature regimes (Rutherford, Mucina, & Powrie, 2006). However, despite this, *M. dolomieu* has not only survived, but also established viable populations and spread throughout the systems into which it was introduced (Van Der Walt, Weyl, Woodford, & Radloff, 2016). Although the initial introduced individuals may have been of admixed stock, the substantial admixture observed among *M. dolomieu* populations in the invaded range may also have resulted from hybridization post introduction (Diedericks, Henriques, von der Heyden, Weyl, & Hui, 2018) as has been observed for *M. dolomieu* introductions elsewhere (Avise et al., 1997; Bagley, Mayden, Roe, Holznagel, & Harris, 2011; Pipas & Bulow, 1998; Whitmore & Butler, 1982; Whitmore & Hellier, 1988). Further, although sampling was conducted away from known angling “hotspots,” *M. dolomieu* are popular angling species and human‐mediated, long‐distance dispersal via intentional stocking cannot be excluded as a mechanism. Such long‐distance (human‐mediated) dispersal events are known to increase population mixing, ultimately increasing the species’ genetic diversity and hence, adaptability to the novel environment (Berthouly‐Salazar et al., 2013).

### 4.3 The influence of sampling design on genetic diversity

Molecular techniques are indispensable tools in invasion biology (Blanchet, 2012; Muirhead et al., 2008), particularly for reconstructing species invasion histories and routes (Estoup & Guillemaud, 2010; Guillemaud et al., 2010, 2015; Wilson, Dormontt, Prentis, Lowe, & Richardson, 2009). However, sampling problems such as the number of native versus invasive populations sampled and the number of individuals sampled per population may hinder the accuracy of the molecular markers to identify the source population (Guillemaud et al., 2010). To date, however, no study has looked at the effect that “sampling locality” may have on each populations’ genetic composition and, hence, genetic diversity. For example, aquatic freshwater species, particularly fish, are often collected from natural lakes or man‐made reservoirs due to the ease of collection and the large number of individuals present. These specific sampling sites, however, often display much lower levels of genetic variability when compared to rivers, as suggested by our results (localities BU and MP in the invasive range). Similarly, a recent study reconstructing the invasion history of the largemouth bass, *Micropterus salmoides*, identified extremely low levels of neutral genetic diversity within invasive populations in lentic environments with limited connectivity (Hargrove, Weyl, & Austin, 2017). Their results revealed that all lentic populations had allele frequencies dominated by a single allele, but that a population sampled from Kowie Weir, located at the end of a 580 km^2^ catchment, was more diverse, suggesting multiple introduction events or hybridization between co‐occurring *Micropterus* species (Hargrove et al., 2017). Thus, choice of sampling locality and, in particular, the degree of isolation are important considerations when assessing the demographic or invasion history of a species.

### 4.4 Management implications

Understanding the introduction history of an invasive species is crucial when wanting to decide on a management strategy for the species in question (Prentis et al., 2009). Our results reveal a complex demographic history for *M. dolomieu*, both within its native USA and invasive SA range. With regard to management in the native range, our data support the management recommendations by Brewer and Orth (2015) that stocking should be guided by a rangewide analysis of genetic variation. In South Africa, eradication of *M. dolomieu* is no longer a feasible option due to the magnitude of the invasion, and the current management strategy is to prevent spread into previously uninvaded catchments by restricting stocking (see Woodford et al., 2017). This is a prudent strategy as the facilitation of strategies that might further increase genetic diversity, thought to assist population establishment, persistence and ultimately local adaptation to novel environments, may increase the fitness of this already highly successful invader. As our study demonstrates the possibility of undocumented *M. dolomieu* introductions into the country, it is imperative that South Africa strictly enforces its current legislation with regard to avoiding new introductions of this already invasive species. In addition, introductions even in river systems that have already been invaded may aid in increasing the genetic fitness of these already highly successful invaders and could facilitate further spread and exacerbate the already considerable impacts on native biota (Van Der Walt et al., 2016).

In conclusion, while studies comparing contemporary genetic variation among native and invasive ranges are valuable (Lozier & Cameron, 2009), incorporating historical DNA is essential for monitoring temporal changes in genetic diversity that are often overlooked in comparisons using only contemporary data (Hansen, 2002; Lozier & Cameron, 2009). Using the smallmouth bass, *M. dolomieu*, as study organism, our results corroborate the idea that genetic variation can indeed change over spatiotemporal scales. Both CI and CN range displayed high levels of admixture and limited population structuring. Although this pattern is not uncommon for invasive species that have been introduced multiple times, our results suggest that various factors may have played a role in shaping the genetic diversity of the CI range.

Our study highlights the importance of including historical DNA; however, caution should be taken when working with historical specimens as the degraded nature of the DNA not only hampers the successful amplification of the specimens (Sefc, Payne, & Sorenson, 2003; Sefc et al., 2007), but also renders it susceptible to genotyping discrepancies. Despite this, we recommend that future studies attempting to infer the demographic history of invasive species should incorporate native historical samples.

## CONFLICT OF INTEREST

None declared.

## DATA ARCHIVING STATEMENT

Data available from the Dryad Digital Repository: https://doi.org/10.5061/dryad.5jf41k5.

## APPENDIX 1

### A detailed description of specimens obtained from various museums, including the specimen origin, collection date, specimen abbreviation corresponding to that used in Table 1, museum responsible for the specimen and its corresponding accession number

**Table:**

Country	State	Sampled locality	Drainage system	Collection date	Specimen abbrev.	Material supplied By	Accession #	Notes
USA	Maryland	Monocacy River	Potomac River	1941	PO_1	ANSP	ANSP 95683	Fry
USA	Maryland	Monocacy River	Potomac River	1941	PO_2	ANSP	ANSP 95683	Fry
USA	Maryland	Monocacy River	Potomac River	1941	PO_3	ANSP	ANSP 95683	Fry
USA	Maryland	Plummer Is., Maryland.	Potomac River	1930	PO_4	NMNH	USNM 284083	Fin snip & bits of gillraker; might have been exposed to arsenic (As), mercury (Hg), lead (Pb)
USA	Virginia	Shenandoah River	Shenandoah River	1934	SH_1	NMNH	USNM 102132	Muscle tissue
USA	Virginia	Shenandoah River	Shenandoah River	1935	SH_2	NMNH	USNM 93780	Muscle tissue
USA	West Virginia	Shenandoah River	Shenandoah River	1936	SH_3	NMNH	USNM 100694	Muscle tissue
USA	Virginia	Shenandoah River	Shenandoah River	1933	SH_4	NMNH	USNM 104928	Muscle tissue
USA	Ohio	Mosquito Creek	Mosquito Creek	1938	MO_1	OSUM	OSUM 3568	Muscle tissue
USA	Ohio	Mosquito Creek	Mosquito Creek	1938	MO_2	OSUM	OSUM 3568	Muscle tissue
USA	Ohio	Auglaize River	Auglaize River	1940	AU_1	OSUM	OSUM 3814	Muscle tissue
USA	Ohio	Auglaize River	Auglaize River	1940	AU_2	OSUM	OSUM 3814	Muscle tissue
USA	Ohio	Auglaize River	Auglaize River	1940	AU_3	OSUM	OSUM 3942	Muscle tissue
USA	Ohio	Pusheta Creek	Auglaize River	1941	AU_4	OSUM	OSUM 4343	Muscle tissue
USA	Ohio	Pusheta Creek	Auglaize River	1941	AU_5	OSUM	OSUM 4343	Muscle tissue
USA	Ohio	Lake Erie	Lake Erie	1941	LE_1	OSUM	OSUM 4272	Muscle tissue
USA	Ohio	Lake Erie	Lake Erie	1941	LE_2	OSUM	OSUM 4272	Muscle tissue
USA	Ohio	Lake Erie	Lake Erie	1941	LE_3	OSUM	OSUM 4272	Muscle tissue
USA	Ohio	White Oak Creek	Ohio River	1930	OH_1	OSUM	OSUM 10834	Muscle tissue
USA	Ohio	White Oak Creek	Ohio River	1930	OH_2	OSUM	OSUM 10834	Muscle tissue
USA	Ohio	White Oak Creek	Ohio River	1930	OH_3	OSUM	OSUM 10834	Muscle tissue
USA	Michigan	Grosse Isle shore, Detroit river	Detroit River	1935	DE_1	UMMZ	UMMZ 243459	Muscle tissue
USA	Michigan	Grosse Isle shore, Detroit river	Detroit River	1935	DE_2	UMMZ	UMMZ 243459	Muscle tissue
USA	Michigan	Grosse Isle shore, Detroit river	Detroit River	1935	DE_3	UMMZ	UMMZ 243459	Muscle tissue
USA	Michigan	Grosse Isle shore, Detroit river	Detroit River	1935	DE_4	UMMZ	UMMZ 243459	Muscle tissue
USA	Michigan	Detroit River	Detroit River	1935	DE_5	UMMZ	UMMZ 243226	Muscle tissue
USA	Michigan	Detroit River	Detroit River	1935	DE_6	UMMZ	UMMZ 243226	Muscle tissue
USA	Michigan	Detroit River	Detroit River	1935	DE_7	UMMZ	UMMZ 243077	Muscle tissue
USA	Michigan	Detroit River	Detroit River	1935	DE_8	UMMZ	UMMZ 243077	Muscle tissue
USA	Michigan	Detroit River	Detroit River	1935	DE_9	UMMZ	UMMZ 243077	Muscle tissue
Canada	Ontario	Detroit River	Detroit River	1940	DE_10	UMMZ	UMMZ 130878	Muscle tissue
Canada	Ontario	Detroit River	Detroit River	1940	DE_11	UMMZ	UMMZ 130878	Muscle tissue
USA	Michigan	Detroit River	Detroit River	1934	DE_12	UMMZ	UMMZ 243009	Muscle tissue
USA	Michigan	Detroit River	Detroit River	1934	DE_13	UMMZ	UMMZ 243009	Muscle tissue
USA	Michigan	Detroit River	Detroit River	1934	DE_14	UMMZ	UMMZ 243009	Muscle tissue
USA	Michigan	Detroit River	Detroit River	1934	DE_15	UMMZ	UMMZ 243009	Muscle tissue
USA	Ontario	Detroit River	Detroit River	1940	DE_16	UMMZ	UMMZ 130896	Muscle tissue
USA	Ontario	Detroit River	Detroit River	1940	DE_17	UMMZ	UMMZ 130896	Muscle tissue
USA	Ontario	Detroit River	Detroit River	1940	DE_18	UMMZ	UMMZ 130896	Muscle tissue
USA	New York	Otselic River	Susquehanna River	1935	SU_1	UMMZ	UMMZ 109652	Muscle tissue
USA	New York	Otselic River	Susquehanna River	1935	SU_2	UMMZ	UMMZ 109652	Muscle tissue
USA	New York	Otselic River	Susquehanna River	1935	SU_3	UMMZ	UMMZ 109652	Muscle tissue
USA	New York	Susquehanna River	Susquehanna River	1935	SU_4	UMMZ	UMMZ 109759	Muscle tissue
USA	New York	Susquehanna River	Susquehanna River	1935	SU_5	UMMZ	UMMZ 109759	Muscle tissue
USA	New York	Trib Rondout River to Hudson River	Hudson River	1936	HU_1	UMMZ	UMMZ 114240	Muscle tissue
USA	New York	Trib Rondout River to Hudson River	Hudson River	1936	HU_2	UMMZ	UMMZ 114240	Muscle tissue
USA	New York	Trib Rondout River to Hudson River	Hudson River	1936	HU_3	UMMZ	UMMZ 114240	Muscle tissue
USA	New York	Trib Rondout River to Hudson River	Hudson River	1936	HU_4	UMMZ	UMMZ 114240	Muscle tissue
USA	New York	Allegheny River	Alleghany River	1937	AL_1	UMMZ	UMMZ 180878	Muscle tissue
USA	New York	Allegheny River	Alleghany River	1937	AL_2	UMMZ	UMMZ 180878	Muscle tissue
USA	New York	Allegheny River	Alleghany River	1937	AL_3	UMMZ	UMMZ 180878	Muscle tissue
USA	New York	Fall Creek, trib. to Cayuga Lake, Etna	Fall Creek	1931	FC_1	UMMZ	UMMZ 94455	Muscle tissue
USA	New York	Fall Creek, trib. to Cayuga Lake, Etna	Fall Creek	1931	FC_2	UMMZ	UMMZ 94455	Muscle tissue
SA	Eastern Cape	Elandsjacht Dam	Krom	2012	KR2	SAIAB	AC09 B425	Muscle tissue
SA	Eastern Cape	Elandsjacht Dam	Krom	2012	KR3	SAIAB	AC09 B955	Muscle tissue
SA	Eastern Cape	Elandsjacht Dam	Krom	2012	KR4	SAIAB	AC09 B875	Muscle tissue
SA	Eastern Cape	Elandsjacht Dam	Krom	2012	KR5	SAIAB	AC09 B992	Muscle tissue
SA	Eastern Cape	Elandsjacht Dam	Krom	2012	KR6	SAIAB	AC09 B994	Muscle tissue
SA	Eastern Cape	Elandsjacht Dam	Krom	2012	KR7	SAIAB	AC09 B977	Muscle tissue
SA	Eastern Cape	Elandsjacht Dam	Krom	2012	KR8	SAIAB	AC09 B960	Muscle tissue
SA	Eastern Cape	Elandsjacht Dam	Krom	2012	KR9	SAIAB	AC09 B964	Muscle tissue
SA	Eastern Cape	Elandsjacht Dam	Krom	2012	KR10	SAIAB	AC09 B982	Muscle tissue
SA	Eastern Cape	Elandsjacht Dam	Krom	2012	KR11	SAIAB	AC09 B978	Muscle tissue
SA	Eastern Cape	Elandsjacht Dam	Krom	2012	KR12	SAIAB	AC09 B971	Muscle tissue
SA	Eastern Cape	Elandsjacht Dam	Krom	2012	KR13	SAIAB	AC09 B997	Muscle tissue
SA	Eastern Cape	Elandsjacht Dam	Krom	2012	KR14	SAIAB	AC09 B970	Muscle tissue
SA	Eastern Cape	Elandsjacht Dam	Krom	2012	KR15	SAIAB	AC09 B984	Muscle tissue
SA	Eastern Cape	Elandsjacht Dam	Krom	2012	KR16	SAIAB	AC09 B963	Muscle tissue
SA	Eastern Cape	Rooikranz Dam	Buffalo River	2014	BU1	SAIAB	OW14‐965	Muscle tissue
SA	Eastern Cape	Rooikranz Dam	Buffalo River	2014	BU2	SAIAB	OW14‐985	Muscle tissue
SA	Eastern Cape	Rooikranz Dam	Buffalo River	2014	BU3	SAIAB	OW14‐979	Muscle tissue
SA	Eastern Cape	Rooikranz Dam	Buffalo River	2014	BU4	SAIAB	OW14‐941	Muscle tissue
SA	Eastern Cape	Rooikranz Dam	Buffalo River	2014	BU5	SAIAB	OW14‐835	Muscle tissue
SA	Eastern Cape	Rooikranz Dam	Buffalo River	2014	BU6	SAIAB	OW14‐828	Muscle tissue
SA	Eastern Cape	Rooikranz Dam	Buffalo River	2014	BU7	SAIAB	OW14‐791	Muscle tissue
SA	Eastern Cape	Rooikranz Dam	Buffalo River	2014	BU8	SAIAB	OW14‐700	Muscle tissue
SA	Eastern Cape	Rooikranz Dam	Buffalo River	2014	BU9	SAIAB	OW14‐798	Muscle tissue
SA	Eastern Cape	Rooikranz Dam	Buffalo River	2014	BU10	SAIAB	OW14‐688	Muscle tissue
SA	Eastern Cape	Rooikranz Dam	Buffalo River	2014	BU11	SAIAB	OW14‐684	Muscle tissue
SA	Eastern Cape	Rooikranz Dam	Buffalo River	2014	BU12	SAIAB	OW14‐808	Muscle tissue
SA	Eastern Cape	Rooikranz Dam	Buffalo River	2015	BU13	SAIAB	OW14‐737	Muscle tissue
SA	Eastern Cape	Rooikranz Dam	Buffalo River	2015	BU14	SAIAB	OW14‐735	Muscle tissue
SA	Eastern Cape	Rooikranz Dam	Buffalo River	2015	BU15	SAIAB	OW14‐742	Muscle tissue
SA	Eastern Cape	Rooikranz Dam	Buffalo River	2015	BU16	SAIAB	OW14‐724	Muscle tissue
SA	Eastern Cape	Rooikranz Dam	Buffalo River	2015	BU17	SAIAB	OW14‐686	Muscle tissue
SA	Eastern Cape	Rooikranz Dam	Buffalo River	2015	BU18	SAIAB	OW14‐797	Muscle tissue
SA	Eastern Cape	Rooikranz Dam	Buffalo River	2015	BU19	SAIAB	OW14‐796	Muscle tissue
SA	Eastern Cape	Rooikranz Dam	Buffalo River	2015	BU20	SAIAB	OW14‐675	Muscle tissue
SA	Eastern Cape	Rooikranz Dam	Buffalo River	2015	BU21	SAIAB	OW14‐702	Muscle tissue
SA	Eastern Cape	Rooikranz Dam	Buffalo River	2015	BU22	SAIAB	OW14‐744	Muscle tissue
SA	Eastern Cape	Rooikranz Dam	Buffalo River	2015	BU23	SAIAB	OW14‐705	Muscle tissue
SA	Eastern Cape	Rooikranz Dam	Buffalo River	2015	BU24	SAIAB	OW14‐782	Muscle tissue
SA	Eastern Cape	Rooikranz Dam	Buffalo River	2015	BU25	SAIAB	OW14‐732	Muscle tissue
SA	Eastern Cape	Rooikranz Dam	Buffalo River	2015	BU26	SAIAB	OW14‐746	Muscle tissue
SA	Eastern Cape	Rooikranz Dam	Buffalo River	2015	BU27	SAIAB	OW14‐756	Muscle tissue
SA	Eastern Cape	Rooikranz Dam	Buffalo River	2015	BU28	SAIAB	OW14‐738	Muscle tissue
SA	Eastern Cape	Rooikranz Dam	Buffalo River	2015	BU29	SAIAB	OW14‐733	Muscle tissue
SA	Eastern Cape	Rooikranz Dam	Buffalo River	2015	BU30	SAIAB	OW14‐739	Muscle tissue
SA	Eastern Cape	Rooikranz Dam	Buffalo River	2015	BU31	SAIAB	OW14‐799	Muscle tissue
SA	Eastern Cape	Rooikranz Dam	Buffalo River	2015	BU32	SAIAB	OW14‐715	Muscle tissue
SA	Eastern Cape	Rooikranz Dam	Buffalo River	2015	BU33	SAIAB	OW14‐704	Muscle tissue
SA	Eastern Cape	Rooikranz Dam	Buffalo River	2015	BU34	SAIAB	OW14‐762	Muscle tissue
SA	Eastern Cape	Rooikranz Dam	Buffalo River	2015	BU35	SAIAB	OW14‐727	Muscle tissue
SA	Eastern Cape	Rooikranz Dam	Buffalo River	2015	BU36	SAIAB	OW14‐690	Muscle tissue
SA	Eastern Cape	Rooikranz Dam	Buffalo River	2015	BU37	SAIAB		Muscle tissue
SA	Eastern Cape	Rooikranz Dam	Buffalo River	2015	BU38	SAIAB		Muscle tissue
SA	Eastern Cape	Rooikranz Dam	Buffalo River	2015	BU39	SAIAB		Muscle tissue
SA	Eastern Cape	Rooikranz Dam	Buffalo River	2015	BU40	SAIAB		Muscle tissue
SA	Eastern Cape	Rooikranz Dam	Buffalo River	2015	BU41	SAIAB		Muscle tissue
SA	Eastern Cape	Rooikranz Dam	Buffalo River	2015	BU42	SAIAB		Muscle tissue
SA	Eastern Cape	Rooikranz Dam	Buffalo River	2015	BU43	SAIAB		Muscle tissue
SA	Eastern Cape	Rooikranz Dam	Buffalo River	2015	BU44	SAIAB		Muscle tissue
SA	Eastern Cape	Rooikranz Dam	Buffalo River	2015	BU45	SAIAB		Muscle tissue
SA	Eastern Cape	Rooikranz Dam	Buffalo River	2015	BU46	SAIAB		Muscle tissue
SA	Eastern Cape	Rooikranz Dam	Buffalo River	2015	BU47	SAIAB		Muscle tissue
SA	Eastern Cape	Rooikranz Dam	Buffalo River	2015	BU48	SAIAB		Muscle tissue

## APPENDIX 2

### The scenario information used in the approximate Bayesian computation (ABC) implemented in DIYABC

#### SCENARIO 1–6

Scenario 1: CI originated from the HN stock, which represents a subsample of the CN populations; Scenario 2: CI originated from CN populations, with both populations being derived from HN (i.e., a more recent introduction event than the one on record); Scenario 3: CI did not originate from either CN or HN population, but rather from an unsampled population; Scenario 4: CI populations represent admixed populations from both CN and HN; Scenario 5: CI populations originate from an admixture event between the sampled HN and an unsampled ghost population; Scenario 6: CI populations originate from an admixture event between the sampled CN populations and an unsampled ghost population.

#### SCENARIO A‐I

Scenario A: Most of the CI individuals and the subsample of SA individuals (CI_S_) are more closely related to one another than to any other population, but originated from HN stock which came from the CN gene pool. Scenario B: Both CI and CI_S_ individuals are closest related to one another, while CN and HN are more closely related to one another. Both invasive (CI and CI_S_) and native (CN and HN) groupings stem from a communal source population. Scenario C, like scenario A, states that CI and CI_S_ are most closely related, originating from the CN population. Both CN and CI + CI_S_ populations, in turn, originating from the HN stock. Scenario D proposes a closer tie between HN and CI_S_. This grouping (HN + CI_S_) along with CI individuals originated from a CN population. In scenario E, the HN and CI_S_ are once again closest related to one another, originating from CN. The Remaining CI individuals along with the HN + CI_S_ + CN grouping originate from an unsampled population. Scenario F supports the STRUCTURE results, and states that HN and CI_S_ are most closely related, while CI and CN are more closely related. Both groupings (HN + CI_S_ and CI + CN) share an unsampled ghost origin. Like scenario F, scenario G groups HN and CI together and CN and CI_S_ together. Both groupings (HN + CI and CN + CI_S_) originate from an unsampled ghost population. Scenario H proposes a closer tie between HN and CI. This grouping (HN + CI) along with CI_S_ individuals originated from a CN population. At last, like scenario H, scenario I suggests a closer tie between HN and CI. This grouping (HN + CI) as well as the CN population each originate from independent introductions from CI_S_.

#### SCENARIO I–III

The following three scenarios were run to test if both introductions (CI and CI_S_) did in fact originate from one source population, that is, USA (CN + HN). Scenario G: Both CI and CI_S_ originated independently from the source population (i.e., multiple introductions from single source). Contrastingly, scenario H suggests that only CI_S_ originated from the source population, with CI originating from CI_S_ (i.e., single introduction). At last, scenario I states that both CI and CI_S_ were founded independently from an unsampled source population, which in turn originated from the source (i.e., multiple introductions, but only a single introduction from the source).
