# Supplementary material for: The ghost of introduction past: Spatial and temporal variability in the genetic diversity of invasive smallmouth bass
Source: Evol Appl. 2018 Jun 25;11(9):1609–29. doi: 10.1111/eva.12652 (PMC6183467; doi:10.1111/eva.12652)
Supplement: Supplementary file 1 [file EVA-11-1609-s001.docx]

**SUPPLEMENTARY MATERIAL**

**Table S1.** Microsatellite loci amplified in the present study with the corresponding primer sequence, reference, size, repetition pattern, optimised annealing temperature, multiplex reaction each locus was part of and dye labels used. Shaded areas represent microsatellite loci that were excluded from the study due to amplification errors.

| **Locus** | **Primer Pair and Sequence** | **Reference** | **Size (bp)** | **Core Repeat** | **Amplified** | **Multiplex #** | **Ta (°C)** | **Dye Label** |
| --- | --- | --- | --- | --- | --- | --- | --- | --- |
| Mdo 1 | F: 5' GCTCTTCCCAGTGGTGAGTC 3' R: 5' ATCTCAGCCCATACCGTCAC 3' | Malloy et al. 2000 | 210 | (GT)_14_ | X |  |  |  |
| Mdo 2 | F: 5' GCCCTTTCATATTGGGACAA 3' R: 5' CTGCTCTGGCGTACATTTCA 3' | Malloy et al. 2000 | 197 | (GT)_14_ | X |  |  |  |
| Mdo 3 | F: 5' AGGTGCTTTGCGCTACAAGT 3' R: 5' CTGCATGGCTGTTATGTTGG 3' | Malloy et al. 2000 | 135 | (CA)_20_ | √ | 1 | 53.9 | 6-FAM |
| Mdo 4 | F: 5' TCTGAACAACTGCATTTAGACTG 3' R: 5' CTAATCCCAGGGCAAGACTG 3' | Malloy et al. 2000 | 142 | (CA)_11_ | √ | 1 | 53.9 | NED |
| Mdo 5 | F: 5' CAGGTTCCCTCTCACCTTCA 3' R: 5' ATGGTCTCACCAGGGACAAA 3' | Malloy et al. 2000 | 200 | (CT)_9_CC(CA)_10_GA(CA)_3_TA(CA)_2_ | √ | 2 | 61.0 | PET |
| Mdo 6 | F: 5' TGAAATGTACGCCAGAGCAG 3' R: 5' TGTGTGGGTGTTTATGTGGG 3' | Malloy et al. 2000 | 150 | (CA)_7_(TA)_4_ | X |  |  |  |
| Mdo 7 | F: 5' TCAAACGCACCTTCACTGAC 3' R: 5' GTCACTCCCATCATGCTCCT 3' | Malloy et al. 2000 | 172 | (CA)_12_ | √ | 1 | 53.9 | VIC |
| Mdo 8 | F: 5' GTGAGGACCAGCCAAAATGT 3' R: 5' GGAAGATTGAGGTCCCAACA 3' | Malloy et al. 2000 | 220 | (CA)_19_ | √ | 3 | 58.3 | NED |
| Mdo 9 | F: 5' TTTGATGGGCGTTTTGTGTA 3' R: 5' GACCGGTCCTGCATATGATT 3' | Malloy et al. 2000 | 126 | (GT)_10_ | √ | 3 | 58.3 | PET |
| Mdo 10 | F: 5' GTGTCTCCGTGTGTTGATGG 3' R: 5' AACACCAGAGGCAAACAAGC 3' | Malloy et al. 2000 | 101 | (GT)_10_ | √ | 3 | 58.3 | VIC |
| Mdo 11 | F: 5' TTGTGGAGAGGGGCATAAAC 3' R: 5' GCATCCTCCCACGTTACCTA 3' | Malloy et al. 2000 | 174 | (GT)_11_GA(GT)_3_ | √ | 3 | 58.3 | 6-FAM |
| Lma21 | F: 5' CAGCTCAATAGTTCTGTCAGG 3' R: 5' ACTACTGCTGAAGATATTGTAG 3' | Colbourne et al. 1996 | 158-182 | (TC)_19_(AC)_11_ | √ | 2 | 61.0 | 6-FAM |
| Lma87 | F: 5' ATGACACAGACTCACCATGC 3' R: 5' CTCCTGCCCATAAATCAGAC 3' | Colbourne et al. 1996 | 118-152 | (AC)_15_A_5_ | X |  |  |  |
| Lma102* | F: 5' CTGTGAAAATGGTGTGAGCG 3' R: 5' AAACACAAAAGTCCACGCAC 3' | Neff et al. 1999 | 88-102 | (GT)_19_ATGTAT(GT)_4_ | √ | 2 | 61.0 | NED |
| Lma117* | F: 5' CCACCAACAGCATGCAGAC 3' R: 5' CATGCCACTCATTGCACTG 3' | Neff et al. 1999 | 194-218 | (GT)_22_ | √ | 2 | 61.0 | VIC |

* Amplified microsatellite loci, but not polymorphic. Hence, both Lma102 and Lma117 were excluded from the dataset.

**Table S2.** Pairwise *F_ST_* values between native and invasive *M. dolomieu* populations based on the two partial mtDNA gene fragments, cytb (below diagonal) and CR (above diagonal). Statistically significant results in bold (*P* < 0.05).

|  |  | **Invasive SA (CI) Localities** | | | | | | | | **Native USA (CN) Localities** | | | | | | | | |
| --- | --- | --- | --- | --- | --- | --- | --- | --- | --- | --- | --- | --- | --- | --- | --- | --- | --- | --- |
|  |  | **BE** | **BR** | **BU** | **DO** | **KO** | **KR** | **MP** | **OL** | **DET** | **HUD** | **LOL** | **NIA** | **ONEI** | **ONEO** | **SAR** | **STL** | **VES** |
| **Contemporary Invasive (CI) SA Localities** | **BE** | 0.000 | **0.070** | **0.070** | **0.075** | **0.068** | **0.069** | **0.093** | **0.086** | - | **0.068** | **0.125** | **0.076** | **0.074** | **0.072** | 0.075 | **0.093** | **0.088** |
|  | **BR** | **0.033** | 0.000 | **0.007** | 0.006 | **0.015** | 0.005 | **0.011** | **0.020** | - | 0.010 | **0.065** | **0.021** | **0.016** | 0.010 | 0.011 | **0.039** | **0.028** |
|  | **BU** | **0.024** | **0.014** | 0.000 | 0.002 | **0.015** | 0.000 | **0.037** | **0.020** | - | 0.008 | **0.063** | **0.020** | **0.014** | 0.009 | 0.009 | **0.037** | **0.026** |
|  | **DO** | **0.016** | **0.016** | **0.020** | 0.000 | **0.018** | 0.003 | **0.034** | **0.020** | - | 0.011 | **0.066** | **0.022** | **0.017** | 0.011 | 0.012 | **0.040** | **0.029** |
|  | **KO** | **0.150** | **0.080** | **0.091** | **0.116** | 0.000 | 0.004 | **0.032** | **0.055** | - | 0.008 | **0.063** | **0.020** | **0.015** | 0.009 | 0.009 | **0.037** | **0.027** |
|  | **KR** | **0.101** | **0.041** | **0.052** | **0.072** | -0.010 | 0.000 | **0.031** | **0.037** | - | 0.000 | **0.059** | **0.013** | 0.007 | 0.000 | 0.000 | **0.031** | **0.019** |
|  | **MP** | **0.025** | 0.005 | **0.017** | **0.015** | **0.121** | **0.076** | 0.000 | **0.057** | - | **0.030** | **0.085** | **0.041** | **0.036** | **0.032** | 0.033 | **0.058** | **0.049** |
|  | **OL** | **0.034** | 0.009 | **0.020** | **0.016** | **0.093** | **0.053** | **0.015** | 0.000 | - | **0.049** | **0.104** | **0.059** | **0.055** | **0.052** | **0.054** | **0.076** | **0.068** |
| **Contemporary Native (CN) USA Localities** | **DET** | 0.023 | 0.014 | 0.020 | 0.008 | **0.149** | 0.091 | 0.010 | 0.015 | 0.000 | - | - | - | - | - | - | - | - |
|  | **HUD** | **0.037** | **0.028** | **0.033** | **0.022** | **0.147** | **0.096** | **0.024** | **0.029** | 0.017 | 0.000 | **0.036** | 0.005 | -0.003 | 0.000 | -0.008 | 0.011 | **0.019** |
|  | **LOL** | **0.034** | **0.025** | **0.031** | **0.020** | **0.144** | **0.093** | **0.022** | **0.026** | 0.015 | -0.006 | 0.000 | **0.042** | **0.038** | **0.062** | 0.015 | 0.001 | **0.079** |
|  | **NIA** | **0.042** | **0.033** | **0.038** | **0.028** | **0.143** | **0.097** | **0.029** | **0.034** | 0.024 | **0.025** | **0.016** | 0.000 | 0.007 | 0.013 | -0.017 | **0.020** | **0.031** |
|  | **ONEI** | **0.026** | **0.018** | **0.024** | **0.012** | **0.132** | **0.083** | **0.014** | **0.019** | 0.006 | **0.016** | **0.019** | 0.010 | 0.000 | 0.007 | -0.001 | 0.016 | **0.025** |
|  | **ONEO** | **0.043** | **0.033** | **0.039** | **0.028** | **0.162** | **0.107** | **0.029** | **0.035** | 0.023 | -0.013 | -0.032 | 0.008 | **0.020** | 0.000 | 0.000 | **0.030** | 0.020 |
|  | **SAR** | **0.028** | **0.019** | **0.025** | **0.013** | **0.144** | **0.090** | **0.015** | **0.020** | 0.007 | 0.011 | 0.008 | -0.008 | 0.002 | -0.003 | 0.000 | -0.004 | 0.021 |
|  | **STL** | **0.038** | **0.029** | **0.035** | **0.024** | **0.139** | **0.093** | **0.026** | **0.030** | 0.019 | **0.027** | **0.021** | 0.002 | 0.008 | 0.009 | -0.007 | 0.000 | **0.049** |
|  | **VES** | **0.059** | **0.049** | **0.054** | **0.043** | **0.172** | **0.121** | **0.045** | **0.050** | 0.042 | 0.019 | -0.003 | 0.010 | **0.031** | -0.035 | 0.018 | 0.017 | 0.000 |

**Table S3.** Genetic diversity measures for all 18 localities (contemporary invasive SA = BE – OL, contemporary native USA = DET – VES, historical native USA = MUS) at nine microsatellite loci: **n** – number of successfully genotyped individuals; **Na** – number of alleles; **AR** – allelic richness following the rarefaction analysis; **H_E_** – expected heterozygosity; **H_O_** – observed heterozygosity; **F_IS_** – inbreeding coefficient. Statistical significant results in bold (*P* < 0.05).

|  | **LOCALITY** | | | | | | | | | | | | | | | | | | | |
| --- | --- | --- | --- | --- | --- | --- | --- | --- | --- | --- | --- | --- | --- | --- | --- | --- | --- | --- | --- | --- |
|  |  |  | **BE** | **BR** | **BU** | **DO** | **KO** | **KR** | **MP** | **OL** | **MUS** | **DET** | **HUD** | **LOL** | **NIA** | **ONEI** | **ONEO** | **SAR** | **STL** | **VES** |
| **Mdo3** | 571 | **n** | 22 | 43 | 48 | 38 | 46 | 15 | 50 | 44 | 52 | 7 | 21 | 20 | 49 | 27 | 10 | 10 | 55 | 14 |
|  | 10 | **Na** | 3 | 3 | 1 | 4 | 4 | 2 | 1 | 3 | 10 | 3 | 3 | 3 | 3 | 3 | 3 | 3 | 3 | 3 |
|  | 2.73 | **AR** | 2.86 | 2.55 | 1.00 | 2.52 | 3.03 | 1.94 | 1.00 | 2.78 | 3.92 | 2.95 | 2.82 | 2.40 | 2.72 | 2.28 | 2.16 | 2.92 | 2.57 | 2.69 |
|  | 0.48 | **H_E_** | 0.62 | 0.58 | 0.00 | 0.48 | 0.64 | 0.37 | 0 | 0.61 | 0.73 | 0.63 | 0.62 | 0.38 | 0.55 | 0.48 | 0.28 | 0.65 | 0.52 | 0.58 |
|  | 0.41 | **H_O_** | 0.27 | 0.60 | 0.00 | 0.39 | 0.65 | 0.33 | 0 | 0.39 | 0.46 | 0.71 | 0.62 | 0.25 | 0.51 | 0.33 | 0.30 | 0.60 | 0.49 | 0.43 |
|  | 0.17 | **F_IS_** | **0.57** | -0.05 | \ | 0.18 | -0.02 | 0.10 | \ | **0.37** | **0.37** | -0.15 | -0.01 | 0.35 | 0.07 | 0.30 | -0.08 | 0.08 | 0.06 | 0.27 |
| **Mdo4** | 565 | **n** | 22 | 43 | 48 | 35 | 46 | 15 | 50 | 44 | 51 | 7 | 20 | 20 | 49 | 26 | 10 | 10 | 55 | 14 |
|  | 17 | **Na** | 2 | 2 | 2 | 3 | 3 | 2 | 1 | 3 | 16 | 3 | 2 | 2 | 4 | 2 | 2 | 2 | 2 | 2 |
|  | 2.30 | **AR** | 1.99 | 1.76 | 1.51 | 1.76 | 2.00 | 1.98 | 1.00 | 2.71 | 4.61 | 2.53 | 2.00 | 2.00 | 2.27 | 2.00 | 2.00 | 1.99 | 1.97 | 1.99 |
|  | 0.41 | **H_E_** | 0.49 | 0.24 | 0.14 | 0.21 | 0.31 | 0.43 | 0.00 | 0.61 | 0.79 | 0.38 | 0.51 | 0.51 | 0.43 | 0.51 | 0.51 | 0.44 | 0.44 | 0.45 |
|  | 0.38 | **H_O_** | 0.23 | 0.28 | 0.15 | 0.17 | 0.28 | 0.47 | 0.00 | 0.45 | 0.37 | 0.43 | 0.50 | 0.70 | 0.43 | 0.42 | 0.80 | 0.40 | 0.60 | 0.21 |
|  | 0.11 | **F_IS_** | **0.54** | -0.15 | -0.07 | 0.18 | 0.09 | -0.08 | \ | **0.25** | **0.53** | -0.13 | 0.02 | -0.38 | 0.01 | 0.17 | -0.64 | 0.10 | -0.38 | 0.54 |
| **Mdo5** | 558 | **n** | 22 | 42 | 48 | 38 | 46 | 15 | 49 | 44 | 44 | 7 | 21 | 20 | 48 | 27 | 10 | 10 | 53 | 14 |
|  | 20 | **Na** | 3 | 3 | 2 | 5 | 4 | 2 | 4 | 5 | 10 | 1 | 5 | 3 | 4 | 3 | 3 | 3 | 4 | 5 |
|  | 2.83 | **AR** | 2.63 | 2.20 | 1.93 | 2.74 | 2.13 | 1.91 | 2.56 | 3.46 | 4.17 | 1.00 | 2.78 | 2.40 | 2.92 | 1.33 | 2.42 | 2.57 | 2.54 | 3.23 |
|  | 0.45 | **H_E_** | 0.51 | 0.52 | 0.38 | 0.59 | 0.41 | 0.33 | 0.57 | 0.68 | 0.75 | 0.00 | 0.44 | 0.54 | 0.55 | 0.07 | 0.47 | 0.43 | 0.39 | 0.56 |
|  | 0.40 | **H_O_** | 0.27 | 0.36 | 0.50 | 0.63 | 0.30 | 0.27 | 0.69 | 0.55 | 0.30 | 0.00 | 0.52 | 0.65 | 0.48 | 0.07 | 0.60 | 0.30 | 0.32 | 0.36 |
|  | 0.14 | **F_IS_** | **0.47** | **0.31** | -0.32 | -0.07 | **0.26** | 0.20 | -0.23 | **0.20** | **0.61** | \ | -0.19 | -0.21 | 0.13 | -0.01 | -0.30 | 0.31 | 0.17 | **0.37** |
|  |  |  |  |  |  |  |  |  |  |  |  | *Table S3 continued on next page* | | | | | | | | |
|  |  |  |  |  |  |  |  |  |  |  |  |  |  |  |  |  |  |  |  |  |
|  |  |  |  |  |  |  |  |  |  |  |  |  |  |  |  |  |  |  |  |  |
|  |  |  |  |  |  |  |  |  |  |  |  |  |  |  |  |  |  |  |  |  |
|  |  |  |  |  |  |  |  |  |  |  |  |  |  |  | *Table S3 continued* | | | | | |
| **Mdo7** | 571 | **n** | 22 | 43 | 48 | 38 | 46 | 15 | 50 | 44 | 52 | 7 | 21 | 20 | 49 | 27 | 10 | 10 | 55 | 14 |
|  | 11 | **Na** | 3 | 4 | 4 | 6 | 4 | 4 | 3 | 4 | 11 | 3 | 5 | 3 | 5 | 3 | 3 | 2 | 3 | 3 |
|  | 3.26 | **AR** | 2.59 | 3.11 | 3.49 | 3.64 | 3.70 | 3.52 | 2.48 | 3.59 | 4.52 | 2.86 | 2.80 | 2.22 | 2.98 | 2.16 | 2.45 | 1.71 | 2.08 | 2.79 |
|  | 0.58 | **H_E_** | 0.47 | 0.65 | 0.69 | 0.7 | 0.75 | 0.72 | 0.43 | 0.73 | 0.79 | 0.56 | 0.57 | 0.51 | 0.59 | 0.51 | 0.54 | 0.19 | 0.51 | 0.59 |
|  | 0.52 | **H_O_** | 0.41 | 0.51 | 0.73 | 0.71 | 0.72 | 0.80 | 0.46 | 0.64 | 0.46 | 0.29 | 0.67 | 0.55 | 0.51 | 0.33 | 0.50 | 0.00 | 0.58 | 0.50 |
|  | 0.10 | **F_IS_** | 0.13 | **0.21** | -0.05 | -0.01 | 0.04 | -0.12 | -0.06 | 0.12 | **0.42** | 0.51 | -0.17 | -0.08 | 0.13 | **0.36** | 0.08 | 1.00 | -0.14 | 0.16 |
| **Mdo8** | 534 | **n** | 22 | 43 | 48 | 37 | 46 | 15 | 50 | 44 | - | 7 | 21 | 20 | 49 | 25 | 10 | 9 | 55 | 14 |
|  | 17 | **Na** | 6 | 4 | 2 | 6 | 4 | 3 | 2 | 7 | - | 5 | 5 | 5 | 6 | 5 | 4 | 3 | 3 | 6 |
|  | 3.78 | **AR** | 4.42 | 3.62 | 1.89 | 3.72 | 2.75 | 2.30 | 1.49 | 3.46 | - | 4.15 | 3.32 | 3.01 | 4.07 | 2.87 | 3.13 | 2.49 | 2.78 | 3.44 |
|  | 0.58 | **H_E_** | 0.80 | 0.73 | 0.33 | 0.72 | 0.54 | 0.35 | 0.13 | 0.63 | - | 0.73 | 0.58 | 0.59 | 0.76 | 0.50 | 0.51 | 0.50 | 0.62 | 0.53 |
|  | 0.53 | **H_O_** | 0.91 | 0.70 | 0.38 | 0.65 | 0.43 | 0.33 | 0.10 | 0.52 | - | 0.71 | 0.38 | 0.60 | 0.76 | 0.48 | 0.40 | 0.44 | 0.71 | 0.50 |
|  | 0.07 | **F_IS_** | -0.14 | 0.05 | -0.13 | 0.10 | 0.20 | 0.05 | 0.24 | **0.17** | - | 0.02 | 0.35 | -0.02 | 0.01 | 0.05 | 0.22 | 0.12 | -0.14 | 0.07 |
| **Mdo9** | 569 | **n** | 22 | 43 | 48 | 38 | 46 | 15 | 50 | 43 | 51 | 7 | 21 | 20 | 49 | 27 | 10 | 10 | 55 | 14 |
|  | 11 | **Na** | 6 | 3 | 3 | 4 | 4 | 3 | 2 | 4 | 10 | 2 | 2 | 2 | 3 | 2 | 2 | 2 | 3 | 2 |
|  | 2.60 | **AR** | 3.51 | 2.68 | 2.24 | 2.27 | 2.37 | 2.87 | 1.78 | 3.08 | 4.27 | 1.97 | 2.00 | 1.90 | 2.09 | 2.00 | 1.97 | 1.97 | 2.08 | 2.00 |
|  | 0.49 | **H_E_** | 0.64 | 0.60 | 0.49 | 0.42 | 0.37 | 0.63 | 0.26 | 0.63 | 0.76 | 0.36 | 0.49 | 0.33 | 0.51 | 0.51 | 0.39 | 0.39 | 0.51 | 0.49 |
|  | 0.46 | **H_O_** | 0.59 | 0.63 | 0.60 | 0.39 | 0.35 | 0.60 | 0.18 | 0.58 | 0.27 | 0.14 | 0.62 | 0.40 | 0.47 | 0.41 | 0.50 | 0.50 | 0.51 | 0.50 |
|  | 0.10 | **F_IS_** | 0.08 | -0.04 | -0.24 | 0.07 | 0.06 | 0.04 | 0.30 | 0.07 | **0.64** | 0.63 | -0.26 | -0.23 | 0.09 | 0.20 | -0.29 | -0.29 | 0.01 | -0.01 |
| **Mdo10** | 572 | **n** | 22 | 43 | 48 | 38 | 46 | 15 | 50 | 44 | 53 | 7 | 21 | 20 | 49 | 27 | 10 | 10 | 55 | 14 |
|  | 6 | **Na** | 2 | 2 | 2 | 2 | 2 | 2 | 2 | 2 | 6 | 1 | 2 | 1 | 2 | 2 | 1 | 1 | 2 | 2 |
|  | 1.71 | **AR** | 1.37 | 1.79 | 1.40 | 1.22 | 1.70 | 1.99 | 1.98 | 1.89 | 2.27 | 1.00 | 1.94 | 1.00 | 1.74 | 1.61 | 1.00 | 1.00 | 1.83 | 2.00 |
|  | 0.22 | **H_E_** | 0.09 | 0.26 | 0.10 | 0.05 | 0.21 | 0.48 | 0.45 | 0.33 | 0.33 | 0.00 | 0.37 | 0.00 | 0.23 | 0.17 | 0.00 | 0.00 | 0.29 | 0.52 |
|  | 0.22 | **H_O_** | 0.09 | 0.30 | 0.10 | 0.05 | 0.24 | 0.33 | 0.46 | 0.27 | 0.23 | 0.00 | 0.29 | 0.00 | 0.27 | 0.19 | 0.00 | 0.00 | 0.31 | 0.86 |
|  | 0.00 | **F_IS_** | -0.02 | -0.17 | -0.04 | -0.01 | -0.13 | 0.31 | -0.03 | 0.17 | **0.32** | \ | 0.24 | \ | -0.14 | -0.08 | \ | \ | -0.07 | -0.70 |
|  |  |  |  |  |  |  |  |  |  |  |  |  |  |  |  | *Table S3 continued on next page* | | | | |
|  |  |  |  |  |  |  |  |  |  |  |  |  |  |  |  |  |  |  |  |  |
|  |  |  |  |  |  |  |  |  |  |  |  |  |  |  |  |  |  |  |  |  |
|  |  |  |  |  |  |  |  |  |  |  |  |  |  |  |  |  |  |  |  |  |
|  |  |  |  |  |  |  |  |  |  |  |  |  |  |  |  | *Table S3 continued* | | | | |
| **Mdo11** | 571 | **n** | 22 | 43 | 48 | 38 | 46 | 15 | 50 | 44 | 52 | 7 | 21 | 20 | 49 | 27 | 10 | 10 | 55 | 14 |
|  | 18 | **Na** | 3 | 2 | 2 | 5 | 4 | 2 | 3 | 3 | 9 | 4 | 2 | 3 | 3 | 3 | 2 | 2 | 2 | 2 |
|  | 2.54 | **AR** | 2.36 | 1.98 | 1.72 | 2.92 | 2.87 | 1.98 | 1.35 | 2.82 | 4.36 | 3.29 | 1.94 | 1.97 | 2.31 | 2.10 | 1.93 | 2.00 | 1.86 | 2.00 |
|  | 0.45 | **H_E_** | 0.54 | 0.44 | 0.22 | 0.50 | 0.63 | 0.43 | 0.08 | 0.61 | 0.80 | 0.66 | 0.37 | 0.27 | 0.53 | 0.40 | 0.34 | 0.52 | 0.31 | 0.52 |
|  | 0.40 | **H_O_** | 0.23 | 0.37 | 0.25 | 0.50 | 0.59 | 0.60 | 0.08 | 0.32 | 0.46 | 0.86 | 0.29 | 0.20 | 0.57 | 0.30 | 0.40 | 0.30 | 0.35 | 0.50 |
|  | 0.16 | **F_IS_** | **0.58** | 0.16 | -0.13 | 0.00 | 0.07 | -0.40 | -0.02 | **0.48** | **0.43** | -0.33 | 0.24 | 0.26 | -0.09 | 0.26 | -0.20 | 0.44 | -0.11 | 0.03 |
| **Lma21** | 564 | **n** | 22 | 42 | 48 | 38 | 46 | 15 | 49 | 44 | 48 | 7 | 21 | 20 | 49 | 27 | 10 | 10 | 54 | 14 |
|  | 24 | **Na** | 7 | 6 | 4 | 8 | 6 | 4 | 3 | 7 | 22 | 4 | 4 | 3 | 5 | 5 | 3 | 5 | 6 | 5 |
|  | 4.15 | **AR** | 4.47 | 3.73 | 2.50 | 4.96 | 3.31 | 3.15 | 2.44 | 4.56 | 5.85 | 3.94 | 3.23 | 2.73 | 3.08 | 3.36 | 2.45 | 3.80 | 3.40 | 3.73 |
|  | 0.69 | **H_E_** | 0.77 | 0.68 | 0.46 | 0.83 | 0.66 | 0.64 | 0.56 | 0.79 | 0.90 | 0.80 | 0.66 | 0.61 | 0.67 | 0.69 | 0.57 | 0.73 | 0.69 | 0.73 |
|  | 0.88 | **H_O_** | 0.68 | 0.81 | 0.52 | 0.95 | 0.87 | 0.87 | 0.90 | 0.93 | 0.79 | 1.00 | 1.00 | 1.00 | 1.00 | 0.96 | 1.00 | 0.80 | 0.89 | 0.86 |
|  | -0.26 | **F_IS_** | 0.12 | -0.19 | -0.13 | -0.15 | -0.32 | -0.37 | -0.61 | -0.18 | **0.12** | -0.27 | -0.54 | -0.67 | -0.50 | -0.40 | -0.82 | -0.11 | -0.28 | -0.18 |
| **Ave. all loci** |  | **n** | 22 | 43 | 48 | 38 | 46 | 15 | 50 | 44 | 50 | 7 | 21 | 20 | 49 | 27 | 10 | 10 | 55 | 14 |
|  |  | **Na** | 4 | 3 | 3 | 5 | 4 | 3 | 3 | 4 | 12 | 3 | 3 | 3 | 4 | 3 | 3 | 3 | 3 | 3 |
|  |  | **AR** | 2.91 | 2.60 | 1.96 | 2.86 | 2.65 | 2.40 | 1.79 | 3.15 | 4.25 | 2.63 | 2.54 | 2.18 | 2.69 | 2.19 | 2.17 | 2.27 | 2.35 | 2.65 |
|  |  | **H_E_** | 0.55 | 0.52 | 0.35 | 0.50 | 0.50 | 0.49 | 0.35 | 0.62 | 0.73 | 0.59 | 0.51 | 0.47 | 0.54 | 0.43 | 0.45 | 0.48 | 0.48 | 0.55 |
|  |  | **H_O_** | 0.41 | 0.51 | 0.40 | 0.49 | 0.49 | 0.51 | 0.41 | 0.52 | 0.42 | 0.59 | 0.54 | 0.54 | 0.55 | 0.39 | 0.56 | 0.42 | 0.53 | 0.52 |
|  |  | **F_IS_** | **0.26** | 0.03 | -0.15 | 0.01 | 0.02 | -0.05 | -0.16 | **0.17** | **0.43** | -0.01 | -0.06 | -0.17 | -0.04 | 0.09 | -0.27 | 0.14 | -0.11 | 0.06 |

**Table S4.** Pairwise *F_ST_* values between contemporary invasive (BE – OL), contemporary native (DET – VES) and historical native (MUS) *M. dolomieu* populations. Values are based on eight microsatellite loci and statistical significant results are indicated in bold (*P* < 0.05).

|  | **BE** | **BR** | **BU** | **DO** | **KO** | **KR** | **MP** | **OL** | **DET** | **HUD** | **LOL** | **NIA** | **ONEI** | **ONEO** | **SAR** | **STL** | **VES** |
| --- | --- | --- | --- | --- | --- | --- | --- | --- | --- | --- | --- | --- | --- | --- | --- | --- | --- |
| **BE** | **—** |  |  |  |  |  |  |  |  |  |  |  |  |  |  |  |  |
| **BR** | **0.231** | **—** |  |  |  |  |  |  |  |  |  |  |  |  |  |  |  |
| **BU** | **0.406** | **0.269** | **—** |  |  |  |  |  |  |  |  |  |  |  |  |  |  |
| **DO** | **0.192** | 0.080 | **0.211** | **—** |  |  |  |  |  |  |  |  |  |  |  |  |  |
| **KO** | **0.229** | **0.088** | **0.237** | **0.066** | **—** |  |  |  |  |  |  |  |  |  |  |  |  |
| **KR** | **0.274** | **0.108** | **0.389** | **0.139** | 0.082 | **—** |  |  |  |  |  |  |  |  |  |  |  |
| **MP** | **0.469** | **0.325** | **0.349** | **0.260** | **0.225** | 0.309 | **—** |  |  |  |  |  |  |  |  |  |  |
| **OL** | **0.146** | 0.064 | **0.269** | **0.101** | **0.071** | **0.097** | **0.274** | **—** |  |  |  |  |  |  |  |  |  |
| **DET** | **0.276** | 0.198 | **0.438** | 0.247 | 0.261 | 0.290 | **0.532** | 0.210 | **—** |  |  |  |  |  |  |  |  |
| **HUD** | **0.278** | 0.180 | **0.397** | **0.233** | **0.226** | **0.278** | **0.461** | 0.159 | **0.094** | **—** |  |  |  |  |  |  |  |
| **LOL** | **0.331** | **0.209** | **0.429** | **0.265** | **0.261** | **0.339** | **0.527** | **0.205** | 0.253 | 0.139 | **—** |  |  |  |  |  |  |
| **NIA** | **0.239** | 0.111 | **0.277** | **0.133** | 0.119 | **0.195** | **0.340** | **0.126** | 0.151 | 0.088 | **0.072** | **—** |  |  |  |  |  |
| **ONEI** | **0.306** | 0.175 | **0.407** | **0.212** | **0.182** | **0.248** | **0.464** | 0.167 | 0.263 | 0.148 | 0.073 | 0.049 | **—** |  |  |  |  |
| **ONEO** | **0.335** | **0.212** | **0.431** | **0.261** | **0.251** | **0.334** | **0.534** | **0.196** | 0.283 | 0.144 | 0.004 | 0.062 | 0.038 | **—** |  |  |  |
| **SAR** | **0.289** | **0.197** | **0.468** | **0.263** | **0.227** | **0.259** | **0.537** | **0.182** | 0.185 | 0.161 | **0.128** | 0.093 | 0.094 | 0.121 | **—** |  |  |
| **STL** | **0.300** | 0.129 | **0.367** | 0.184 | 0.157 | **0.219** | **0.402** | **0.157** | 0.218 | 0.122 | **0.102** | 0.041 | 0.037 | **0.098** | **0.129** | **—** |  |
| **VES** | **0.244** | 0.140 | **0.336** | 0.163 | 0.158 | 0.184 | **0.374** | 0.130 | 0.188 | 0.087 | 0.123 | 0.040 | 0.065 | 0.103 | 0.131 | 0.086 | **—** |
| **MUS** | **0.171** | **0.170** | **0.315** | **0.180** | **0.191** | **0.224** | **0.382** | **0.123** | 0.158 | 0.160 | **0.185** | **0.148** | 0.191 | **0.189** | **0.168** | **0.196** | **0.143** |

**Table S5.** The posterior probabilities (direct approach; logistic approach) for each of the competing scenarios conducted in the first (1 - 6), second (A - I) and third (i - iii) ABC analysis. The 95% confidence intervals are indicated in brackets. Type I and Type II error rates are indicated for the scenario with the best support. Scenario details can be found in Appendix 2.

| **Analysis** | **Scenario** | **Posterior probability** | **Type I error** | **Type II error** |
| --- | --- | --- | --- | --- |
| 1 | 1 | 0.0300 [0.0000,0.1795] ; 0.0017 [0.0006,0.0028] |  |  |
| 1 | **2** | 0.4940 [0.0558,0.9322] ; 0.9458 [0.9300,0.9616] | 0.433 | 0.134 [0.060, 0.252] |
| 1 | 3 | 0.0360 [0.0000,0.1993] ; 0.0022 [0.0007,0.0037] |  |  |
| 1 | 4 | 0.1420 [0.0000,0.4480] ; 0.0348 [0.0223,0.0472] |  |  |
| 1 | 5 | 0.2420 [0.0000,0.6174] ; 0.0134 [0.0090,0.0178] |  |  |
| 1 | 6 | 0.0560 [0.0000,0.2575] ; 0.0022 [0.0010,0.0033] |  |  |
| 2 | A | 0.0660 [0.0000,0.2836] ; 0.0697 [0.0000,0.4869] |  |  |
| 2 | B | 0.1220 [0.0000,0.4089] ; 0.0037 [0.0000,0.4438] |  |  |
| 2 | **C** | 0.1280 [0.0000,0.4208] ; 0.7495 [0.6046,0.8943] | 0.302 | 0.044 [0.011, 0.114] |
| 2 | D | 0.0240 [0.0000,0.1582] ; 0.0006 [0.0000,0.4419] |  |  |
| 2 | E | 0.0140 [0.0000,0.1170] ; 0.0000 [0.0000,0.4415] |  |  |
| 2 | **F** | 0.3140 [0.0000,0.7208] ; 0.1496 [0.0000,0.6184] | 0.392 | 0.050 [0.025, 0.095] |
| 2 | G | 0.0840 [0.0000,0.3271] ; 0.0005 [0.0000,0.4418] |  |  |
| 2 | H | 0.0560 [0.0000,0.2575] ; 0.0002 [0.0000,0.4416] |  |  |
| 2 | I | 0.1920 [0.0000,0.5372] ; 0.0262 [0.0000,0.4600] |  |  |
| 3 | i | 0.3200 [0.0000,0.7289] ; 0.2655 [0.2495,0.2815] |  |  |
| 3 | ii | 0.2960 [0.0000,0.6961] ; 0.2700 [0.2543,0.2856] |  |  |
| 3 | **iii** | 0.3840 [0.0000,0.8103] ; 0.4646 [0.4476,0.4816] | 0.625 | 0.227 [0.186, 0.268] |


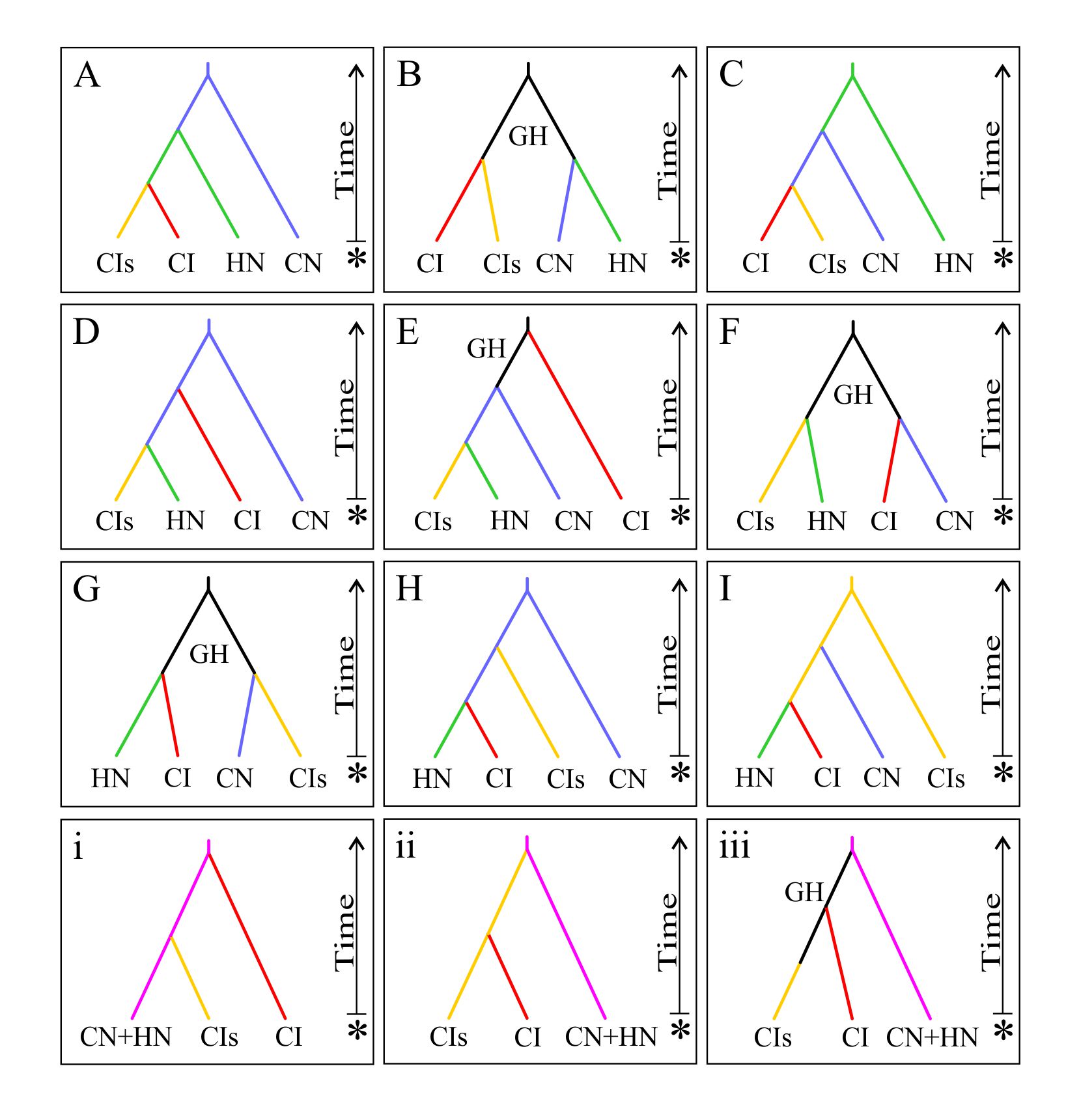


Figure S1. Additional introduction scenarios (multiple introductions: A – I; introductions from a single source: i - iii) as described in Appendix 2 and implemented in DIYABC. CI – contemporary invasive SA (red), CI_S_ – contemporary invasive SA sub-population (yellow), CN – contemporary native USA (blue), HN – historical native USA (green), CN+HN – single USA source population (pink), GH – unsampled ghost population (black). Time in generations is indicated by the arrow, with the present indicated by an asterisk.


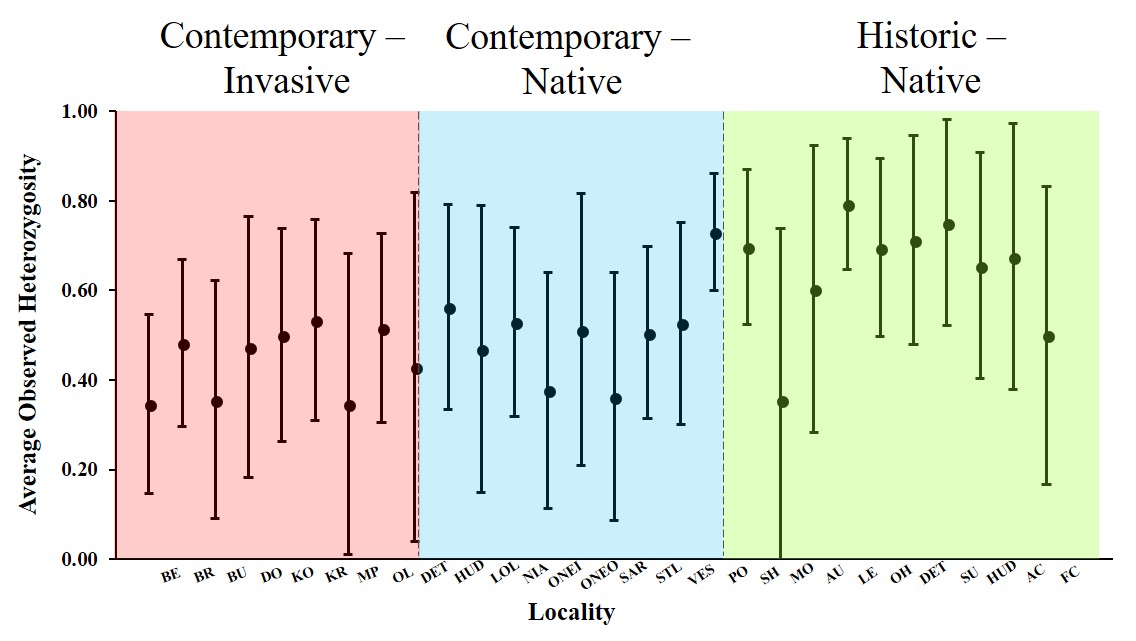
Figure S2. Average variation (± SD) in observed heterozygosity (H_O_) among populations between the three groups (contemporary invasive – CI, contemporary native – CN, historical native – HN).

**REFERENCES**

Colbourne J. K., Neff B. D., Wright J. M., & Gross M. R. (1996). DNA fingerprinting of bluegill sunfish (*Lepomis macrochirus*) using (GT) n microsatellites and its potential for assessment of mating success. *Canadian Journal of Fisheries and Aquatic Sciences*, *53*, 342-349.

Malloy T. P., Van Den Bussche Jr. R. A., Coughlin W. D., & Echelle A. A. (2000). Isolation and characterization of microsatellite loci in smallmouth bass, Micropterus dolomieu (Teleostei: Centrarchidae), and cross-specific amplification in spotted bass, M. punctulatus. *Molecular Ecology, 9*, 191-195.

Neff B. D., Fu P., & Gross M. R. (1999). Microsatellite evolution in sunfish (Centrarchidae). *Canadian Journal of Fisheries and Aquatic Sciences*, *56*, 1198-1205.
